# Dose-dependent effects of camel milk on immune function and metabolic health in weaning rats

**DOI:** 10.1038/s41598-026-35775-0

**Published:** 2026-02-03

**Authors:** Alyaa Farid, Mahy Mohamed, Maryam Amr, Gehan Safwat

**Affiliations:** 1 Biotechnology Department, Faculty of Science, Cairo University, Giza, Egypt; 2 Faculty of Biotechnology, October University for Modern Sciences and Arts, Giza, Egypt

**Keywords:** Breastfeeding, Camel milk, Immunization, Bone health, Immune response, Immunology, Health care

## Abstract

Breastfeeding cannot fulfill an infant’s nutritional needs beyond six months, necessitating the introduction of alternative milk sources. Camel milk has emerged as a promising candidate due to its rich profile of nutrients and immunomodulatory properties. This study evaluated the dose-dependent effects of camel milk on general health and immune response in post-weaning rats, with particular attention to sex-specific differences. Male and female rats were divided into: control (GI), and four treatment groups receiving 2.4 mL (GII), 3.4 mL (GIII), 4.4 mL (GIV), or 5.4 mL (GV) of camel milk daily for six weeks. Serum biochemical parameters, including lipid profile, liver and kidney function markers, and immunological responses were assessed before and after immunization with sheep red blood cells. While higher doses (4.4–5.4 mL) significantly enhanced immune response and bone health, they concurrently elevated liver and kidney function parameters. The 3.4 mL dose balanced benefits, showing significant immune enhancement and bone health improvement without adverse metabolic effects. These findings demonstrated that camel milk (3.4 mL for rats/473 mL for infants) safely enhanced immune function, while higher doses risk metabolic stress. The results supported camel milk’s potential as a nutritional supplement during weaning but emphasized the importance of dose regulation.

**Supplementary Information:**

The online version contains supplementary material available at 10.1038/s41598-026-35775-0.

## Introduction

Regarding newborns, breastfeeding offers the best and most comprehensive nutrition. The health concerns associated with the health concerns associated with infant formula are increasingly well-documented^1^, whereas the advantages of breastfeeding for both the mother and the child have long been acknowledged^2^. Breastfeeding protects against infectious diseases throughout infancy^3^, and it has been linked to long-term advantages in several areas, including cardiovascular disease^4^, cognitive development^5^, and allergic reactions^6^. Even in developed countries, breastfeeding significantly benefits newborns and young children^7^. The transition from exclusive breastfeeding to solid foods, known as weaning^8^, is a critical period where proper nutrition is essential for a child’s optimal growth and development^9,10^. Malnutrition occurs sometimes in children throughout their rapid developmental stage, which might affect their immune system and general health in the long run^11^. When breastfeeding is not possible, mothers often turn to alternative milk sources, such as cow, buffalo, goat, or camel milk^12^.

In desert areas, camels are significant dairy animals, and humans depend on their milk as a source of nutrition. In rural environments, camel milk can be taken either fresh, unheated, or at different levels of sourness. Camel milk contains an intricate blend of fats, amino acids, lactose, vitamins, minerals, and other ingredients. Several factors, including the time of the year and physiological stage, are responsible for the significant diversity in camel milk’s components^13^. The nutritional value and suitability of camel milk for specific dietary needs depends on its distinct compositional similarities and differences with human milk^14^. Human milk is unique in its high lactose and low protein, fat, and mineral content, making it the ideal food for a baby’s growth^15^. It is also distinguished by a high oligosaccharide concentration, essential for enhancing the immunological responses and encouraging the proliferation of advantageous gut microbiota^16^. However, camel milk shares several characteristics with human milk, including a reduced level of saturated fatty acids (SFA) and the absence of β-lactoglobulin (an allergy present in cow’s milk)^17^. Minerals like Fe and Zn are abundant in camel milk. It is a possible substitute for people with dairy allergies because it has a higher monounsaturated fatty acids (MUFA) content than human milk^17,18^.

Camel milk, sometimes known as “white desert gold”, is an essential part of the diet of children and adults in the Arabian Gulf region^19^. According to Mirmiran et al.^20^, camels are biologically capable of producing four to thirty liters of milk per day, even in the face of harsh environmental factors such as high temperatures, limited grazing, and water scarcity. With a maximum breastfeeding duration of two to three months, the camel milk lactation phase lasts nine to eleven months. The scientific community has taken notice of camel milk’s potential therapeutic benefits for adults, particularly in treating diabetes mellitus (type I and type II) and cardiovascular disease^20^. It has been suggested that the special constitution of camel milk, which consists of unsaturated fatty acids, vitamins, minerals, and insulin-like proteins, will improve heart health and glycemic management^21^.

Based on established pediatric nutrition models, previous research has directly compared the health impacts of different milk types (cow, buffalo, goat, and camel) in weaning rats^12,22^. These investigations employed a standardized dose of 3.4 mL/day, which is the direct equivalent of the WHO recommendation for 19-month-old children (473.1 mL/day), based on validated conversion factors where one human year equals 13.5 rat days^23^. This dose was administered to 3-week-old rats, a developmental stage analogous to a 19-month-old human infant^12,22,24^. The collective findings consistently demonstrated that camel milk offers superior benefits for early-weaned subjects compared to other milk types^12,22,24^. Specifically, camel milk supplementation enhanced immune function by promoting B-cell activation and elevating anti-SRBCs antibody titers, while concurrently reducing oxidative stress and spleen inflammation post-immunization^12^. Critically, these advantages were achieved without any adverse effects on kidney, liver, or bone health^22^. Researchers attribute these unique health-promoting properties to camel milk’s distinct nutritional profile, characterized by a lower fat and lactose content, higher concentrations of essential vitamins and minerals, and a richer abundance of polyunsaturated (PUFA) and monounsaturated fatty acids (MUFA)^12,22,24^.

While the beneficial properties of camel milk are established, the critical question of its dose-dependent efficacy and safety remains largely unexplored. Therefore, this study was designed to systematically evaluate a spectrum of camel milk doses, from sub-optimal (2.4 mL) to supra-physiological (4.4 and 5.4 mL), against the WHO-recommended equivalent (3.4 mL) in a weaning rat model. We comprehensively examined the effects of these doses on serum vitamin and mineral levels, kidney and liver function parameters, and the immune response following immunization with SRBCs to assess cytokine and immunoglobulin production. The study included both male and female Sprague Dawley rats to account for sex-specific differences, as sex is a key biological variable known to significantly influence immune system performance^25^. We hypothesized that camel milk administration would exert dose-dependent effects on immune function and metabolic health in weaning rats. We further hypothesized that a WHO-equivalent dose of camel milk would optimize immunological and metabolic outcomes without inducing hepatic or renal stress.

## Materials and methods

### Compositional analysis of camel milk

Camel milk was obtained from she camels (4th-9th week following delivery) at Marsa Matrouh station, with lactation stages monitored according to Animal Production Research Institute (APRI) protocols. Analysis of the milk major components (water content, fats, protein, and lactose) was performed by an infrared spectrometry-based Milkoscan^®^ analyser. Lipid extraction from milk samples was performed following the method described by Liu et al.^26^.

### Fatty acids profile analysis

Determining the fatty acids profile in milk’s fat typically consists of lipid extraction, transesterification and gas chromatography. The study employed an optimized method by Liu et al.^26^ that eliminates the lipid extraction step through direct methylation of centrifugally separated milk fat. After centrifugation of camel milk (14 mL) at 3000xg for 25 min at 4 °C, the top fat layer was separated and methylated by mixing with 2.4 mL of KOH (2 M) followed by 25 min of incubation at 50 °C. A capillary-type gas chromatography column with helium carrier gas was used for profiling fatty acids.

### Amino acids profile analysis

For determination of amino acids, casein was precipitated from skimmed milk using acetic acid (0.01 mol/L) at pH 4.5 to 4.6. After three cycles of water washing, the precipitate was freeze-dried. Under vacuum, HCl (6 mol/L) was used to hydrolyze the acid casein (20–30 mg) for twenty-four hours at 110 °C. A model Liquimat III amino acid analyzer (Kontron Instruments AG, Zurich) was used for the analysis of amino acids in the hydrolysate^27^. Different minerals [phosphorus (P), sodium (Na), potassium (K) and calcium (Ca)] were determined by inductively coupled plasma-optical emission spectrometry (ICP-OES) technique.

### Vitamin content analysis

Water soluble vitamins such as vitamin C and vitamin B complex (B1, B3, B5 and B6) were determined using reversed phase- high performance liquid chromatography (RP-HPLC) with UV detection (λ_em_ at 246 nm) employing a Thermo Scientific Hypersil BDS C18 column (100 × 4.6 mm, 3 μm) with a mobile phase of (A): hexane-1-sulfonic acid Na (5.84 mM): acetonitrile (95:5, v/v) with triethylamine (0.1%) and (B): hexane-1-sulfonic acid Na (5.84 mM):acetonitrile (50:50) with triethylamine (0.1%). pH of both mobile phases was adjusted to 2.5 by orthophosphoric acid (1M)^28^.

Fat soluble vitamins such as vitamin A, vitamin E and vitamin D_3_ were measured in camel milk by the method of Guneser and Yuceer^29^. Milk samples (2 mL) were combined with 50% methanolic KOH (2 mL), and the mixture was vortexed for 1 min to accomplish saponification. After 20 min of heating (80 °C), the mixture was allowed to cool then extracted by n-hexane. After collecting and evaporating the organic phase, it was re-dissolved in acetonitrile/methanol (85:15, v/v). The Agilent1260 infinite HPLC Series, equipped with a Kinetex XB-C18 column (5 μm, 4.6 × 100 mm, Phenomenex, USA) and a UV detector, was used for HPLC analysis. With a flow rate of 1 mL/min, the extract (20 uL) was eluted using acetonitrile/methanol (75:25, v/v), for vitamins A and D3; and (70:30, v/v) for vitamin E. Vitamin A peaks were measured at 325 nm, vitamin E peaks at 292 nm, and vitamin D_2_ peaks at 264 nm. The identification of vitamins was done by examining their retention times to those of the standards. The Agilent software data analysis system was used to quantify the vitamins using a calibration curve.

### Phospholipids analysis

The phospholipid content in the camel milk sample was quantified using Phosphorus-31 Nuclear Magnetic Resonance (³¹P NMR) spectroscopy. Analysis was performed at an operating frequency of 162 MHz on a Bruker Avance III™ HD 400 MHz spectrometer (Bruker BioSpin, USA), following the established procedure of Wei et al.³⁰. Phospholipid classes were identified by comparing their characteristic chemical shifts against an internal standard of Triphenyl phosphate (δ = −17.8 ppm). The resulting ³¹P NMR spectra were processed and analyzed using the Mnova Software Suite (Mestrelab Research, Spain).

### Experimental design

The study employed a total of fifty Sprague Dawley rats, comprising equal numbers of males and females (*n* = 25 per sex), to evaluate potential sex-dependent variations in immune response^25^. Three-week-old rats (weighing 70–80 g) were sourced from the National Cancer Institute (Cairo, Egypt). Animals were housed under controlled environmental conditions (temperature: 22 ± 2 °C; 12-hour light/dark cycle) and provided with ad libitum access to a standard laboratory diet (composition: 18% crude protein, 5% crude oil, 54% carbohydrates) and water. All experimental procedures were conducted in strict compliance with the ARRIVE guidelines and received prior approval from the Institutional Animal Care and Use Committee of October University for Modern Sciences and Arts.

The camel milk doses were selected based on a translational approach. The WHO-recommended intake for 19-month-old children (473.1 mL/day) was converted to a rat-equivalent dose (3.4 mL/day) using the established conversion factor where one human year equals 13.5 rat days^12,22,23,31^. To establish a dose-response relationship, this WHO-equivalent dose was bracketed by both lower and higher doses.

Within each sex, rats were randomly allocated into five experimental groups (*n* = 5 per group per sex):

- GI: Control group, receiving a normal diet.
- GII: Low-dose group, administered 2.4 mL of camel milk daily.
- GIII: Recommended-dose group, administered 3.4 mL (WHO-equivalent) of camel milk daily.
- GIV: High-dose group, administered 4.4 mL of camel milk daily.
- GV: Very high-dose group, administered 5.4 mL of camel milk daily.

Treatments were administered once daily via oral gavage for a total duration of six weeks. Where all animals were monitored daily for general health, signs of distress, mortality, and the presence of clinical symptoms. Body weight was measured to track growth performance and overall well-being. To assess the immune response, all animals were immunized via intraperitoneal injection with a 0.5 mL suspension of 10% SRBCs on day 35 of the study. At the end of the 6-week intervention, terminal blood collection was performed under deep anesthesia induced by an intraperitoneal injection of sodium pentobarbital (80 mg/kg), followed by cardiac puncture to ensure euthanasia. Blood samples were allowed to clot for 2 h at room temperature, and serum was subsequently separated by centrifugation at 1500 rpm for 15 min. The resulting serum aliquots were stored at −80 °C until analysis. To minimize bias, all laboratory analyses were conducted by investigators blinded to the treatment group assignments. The health and well-being of all animals were monitored through daily clinical observations.

The selection of higher camel milk doses (4.4 mL and 5.4 mL) was motivated by a physiological rationale beyond simple United States Department of Agriculture (USDA) equivalency. These doses, equivalent to 612 mL and 751 mL for a 19-month-old child, were designed to model real-world scenarios of high milk consumption. This includes its use as a primary nutritional source in pastoralist communities^19^ and in nutritional rehabilitation protocols for catch-up growth, where energy and nutrient demands are substantially elevated^32^. Furthermore, administering these supra-physiological doses (with 5.4 mL exceeding recommendations by > 25%) was essential to rigorously test the upper safety limit of camel milk intake and identify potential thresholds for metabolic stress on hepatic and renal systems.

### Preparation of SRBCs suspension

Fresh blood was drawn from the sheep’s external jugular vein and combined in a 1:1 ratio with Alsever’s solution [dextrose (2%), Na citrate (0.8%), citric acid (0.055%), and Na chloride (0.42%)] to create the SRBCs suspension for immunization. After the plasma was separated from the blood by centrifuging it for 15 min at 1500 rpm, the SRBCs pellet was rinsed three times with 0.9% saline. For animal immunization, SRBCs were suspended in saline at a concentration of 10%. According to Van Loveren et al.^33^, 10% SRBCs (0.5 mL) suspension was administered intraperitoneally to each rat.

### Analysis of serum minerals and vitamins

Serum levels of key biomarkers related to bone metabolism were quantified. Phosphorus (P) concentration was determined using a commercial rat-specific ELISA kit (MBS3809130, MyBioSource, USA). Total calcium (Ca) concentration, representing both protein-bound and free ionic calcium, was measured on a cobas^®^ c 702 clinical analyzer (Rotkreuz, Switzerland) using the 5-nitro-5′-methyl-BAPTA technique^34^. The physiologically active ionized calcium fraction was calculated indirectly using the established formula from Costa et al.^35^. Vitamin D was assessed by measuring its major metabolites. The storage form, calcifediol (25-hydroxyvitamin D3), was analyzed using a rat-specific ELISA kit (MBS261766, MyBioSource, USA). The active hormonal form, calcitriol (1,25-dihydroxyvitamin D3), was quantified with a corresponding rat-specific ELISA kit (MBS1609064, MyBioSource, USA).

### Assessment of kidney and liver functions

Rat’s ELISA kits were used to measure serum levels of alanine aminotransferase (ALT) and aspartate aminotransferase (AST) (MBS269614 and MBS264975, respectively; MyBioSource, USA). Rat’s urea and creatinine ELISA kits (MBS2600001 and MBS3809095, respectively) were used to test kidney function parameters.

### Assessment of cytokines and Immunoglobulin levels BI and AI

Serum levels of cytokines (IL-1β, IL-6, IL-17 and IL-18) and immunoglobulin (IgM and IgG) were measured by rat’s ELISA kits (ab100767, ab234570, ab214028, ab213909, ab157738 and ab189578, respectively; Abcam, USA) according to the manufacturer’s protocols.

### Histopathological analysis

Upon termination of the experiment, tissue samples from the spleen, liver, and kidney were immediately collected from all animals. Tissues were fixed in 10% neutral buffered formalin for 48 h, processed through a graded series of ethanol and xylene, and embedded in paraffin blocks. Sections of 4–5 μm thickness were cut using a rotary microtome and stained with standard hematoxylin and eosin (H&E) stain for histological examination.

### Statistical analysis

The sample size of *n* = 10 per group (*n* = 5 per sex) was determined based on established precedent in preclinical nutritional studies and the previous work with identical endpoints^22^, which has demonstrated that this size provides sufficient statistical power (> 80%) to detect significant differences in metabolic and immunological parameters while adhering to the ethical principle of reducing animal numbers. All data were presented as mean ± standard deviation (SD). The dose-dependent effects were evaluated using a One-Way Analysis of Variance (ANOVA) followed by a post hoc test for comparisons between multiple groups. To address the specific effects of camel milk, each treatment group (GII, GIII, GIV, GV) was compared to the control group (GI). Furthermore, to assess the impact of lower and higher doses, groups GII, GIV, and GV were also compared to the WHO-recommended equivalent dose group (GIII). To simplify the interpretation of complex, multi-parameter data and mitigate overcrowding in figures, composite Z-scores for weight performance, bone health, vitamin D metabolism, liver function, kidney function, lipid profile, pro-inflammatory response and humoral immunity were calculated by standardizing individual animal values to the mean and standard deviation of the sex-matched control group. In accordance with enhancing biological interpretation, the results were reported with the mean difference, 95% confidence interval (CI), and effect size (Cohen’s d), with |d| ≥ 0.8 considered a large effect. A p-value of less than 0.05 was considered statistically significant. All analyses were conducted with the investigators blinded to the treatment groups.

## Results

### Analysis of camel milk

**Fig. 1:**
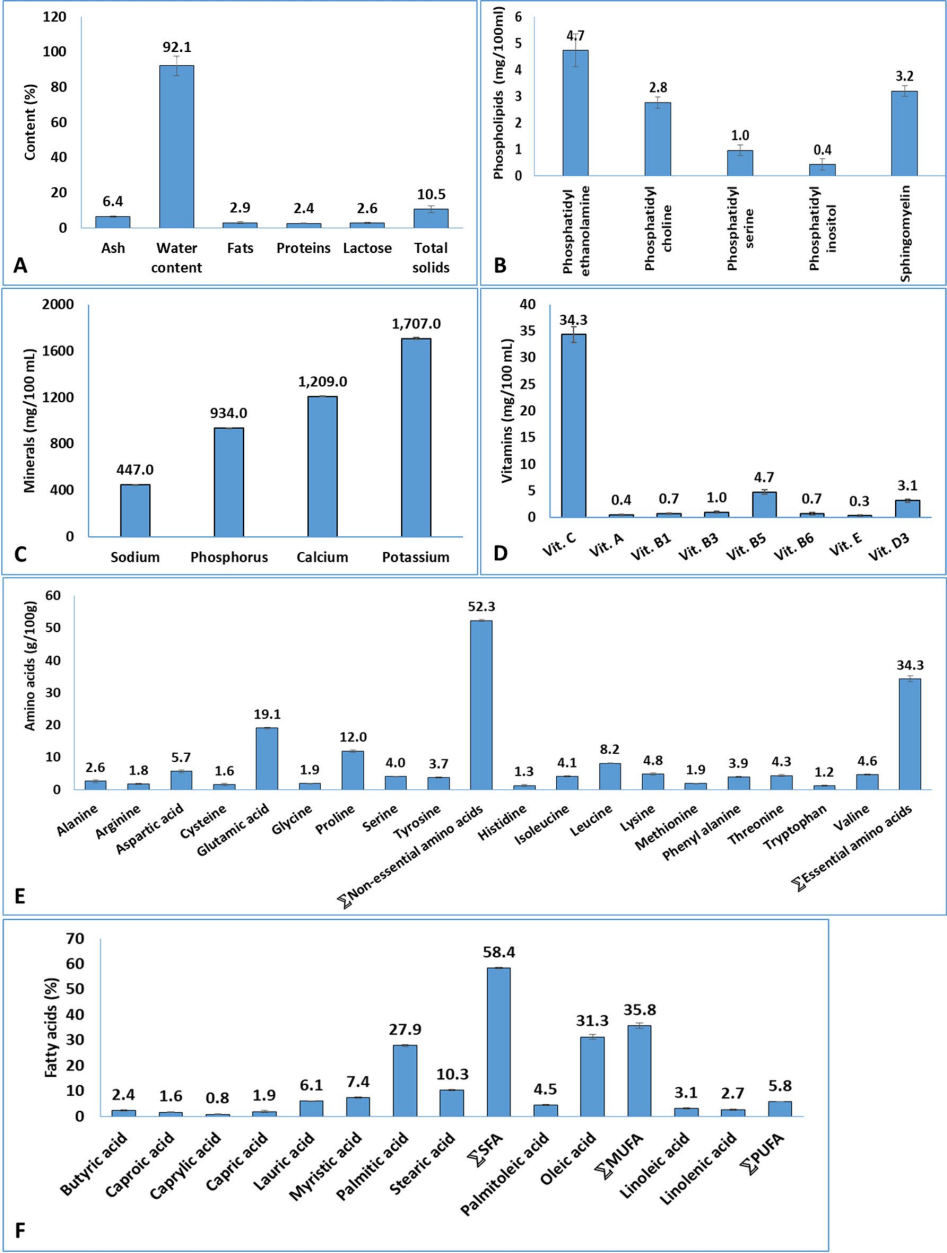
analysis of camel milk showing (**A**) major components (%), (**B**) Phospholipids (mg/100 mL), (**C**) minerals (mg/100 mL), (**D**) vitamins (mg/100 mL), (**E**) amino acids (g/100 g), and (**F**) fatty acids (%). Results were expressed as mean (three samples) ± SD.

The comprehensive analysis revealed camel milk to be composed primarily of water (92.1%), with substantial amounts of ash (6.4%), fats (2.9%), proteins (2.4%), and lactose (2.6%) (Fig. 1A). Phospholipid profiling demonstrated a distinct distribution pattern, with phosphatidyl ethanolamine being the most abundant (4.7 mg/100 mL), followed by sphingomyelin (3.2 mg/100 mL), phosphatidyl choline (2.8 mg/100 mL), phosphatidyl serine (1.0 mg/100 mL), and phosphatidyl inositol (0.4 mg/100 mL) (Fig. 1B).

Mineral analysis identified potassium (K, 1707.0 mg/100 mL) and calcium (Ca, 1209.0 mg/100 mL) as the predominant minerals, accompanied by significant quantities of phosphorus (P, 934.0 mg/100 mL) and sodium (Na, 447.0 mg/100 mL) (Fig. 1C). Vitamin profiling detected both water-soluble and fat-soluble vitamins, with vitamin C (34.3 mg/100 mL) being the most concentrated water-soluble vitamin. The B-vitamin complex showed varying concentrations: B5 (4.7 mg/100 mL) > B3 (1.0 mg/100 mL) > B1 = B6 (0.7 mg/100 mL each). Among fat-soluble vitamins, vitamin D3 (3.1 mg/100 mL) was most abundant, followed by vitamin A (0.4 mg/100 mL) and vitamin E (0.3 mg/100 mL) (Fig. 1D).

Amino acid analysis revealed a total non-essential amino acid content of 52.3 g/100 g, dominated by glutamic acid (19.1 g/100 g), proline (12.0 g/100 g), and aspartic acid (5.7 g/100 g), with lower but significant levels of serine (4.0 g/100 g), tyrosine (3.7 g/100 g), alanine (2.6 g/100 g), glycine (1.9 g/100 g), arginine (1.8 g/100 g), and cysteine (1.6 g/100 g) (Fig. 1E). Essential amino acids totaled 34.3 g/100 g, with leucine (8.2 g/100 g) being most prevalent. Other essential amino acids showed comparable levels: lysine (4.8 g/100 g) > valine (4.6 g/100 g) > threonine (4.3 g/100 g) > isoleucine (4.1 g/100 g) > phenylalanine (3.9 g/100 g) > methionine (1.9 g/100 g) > histidine (1.3 g/100 g) > tryptophan (1.2 g/100 g).

Fatty acid profiling demonstrated a predominance of saturated fatty acids (SFAs, 58.4%), with palmitic acid (27.9%) as the major constituent, followed by stearic acid (10.3%), myristic acid (7.4%), and lauric acid (6.1%). Minor SFAs included butyric acid (2.4%), capric acid (1.6%), caprylic acid (0.8%), and others. Monounsaturated fatty acids (MUFAs) accounted for 35.8% of total fatty acids, primarily comprising oleic acid (31.3%) and palmitoleic acid (4.5%). Polyunsaturated fatty acids (PUFAs, 5.8%) consisted of linoleic acid (3.1%) and α-linolenic acid (2.7%) (Fig. 1F).

### General health, mortality, and growth performance

Camel milk supplementation was well-tolerated at lower doses. No mortality was observed in any group during the study. Furthermore, no adverse clinical signs, such as diarrhea, lethargy, or piloerection, were noted in any group during the study. Analysis of growth parameters revealed a biphasic, dose-dependent effect. The WHO-equivalent dose (GIII) significantly enhanced growth, as evidenced by increased final body weight and weight gain in both sexes compared to the control. In contrast, the supra-physiological doses (GIV, 4.4 mL and GV, 5.4 mL) induced a significant suppression of growth, with a more pronounced effect observed in female rats. No significant differences in food intake were detected between any of the treatment groups and the control, indicating that the growth suppression at high doses was not due to reduced caloric consumption but likely a metabolic consequence of the intervention. Detailed data on body weight was provided in supplementary materials (tables S2, S3 and S4).

**Fig. 2:**
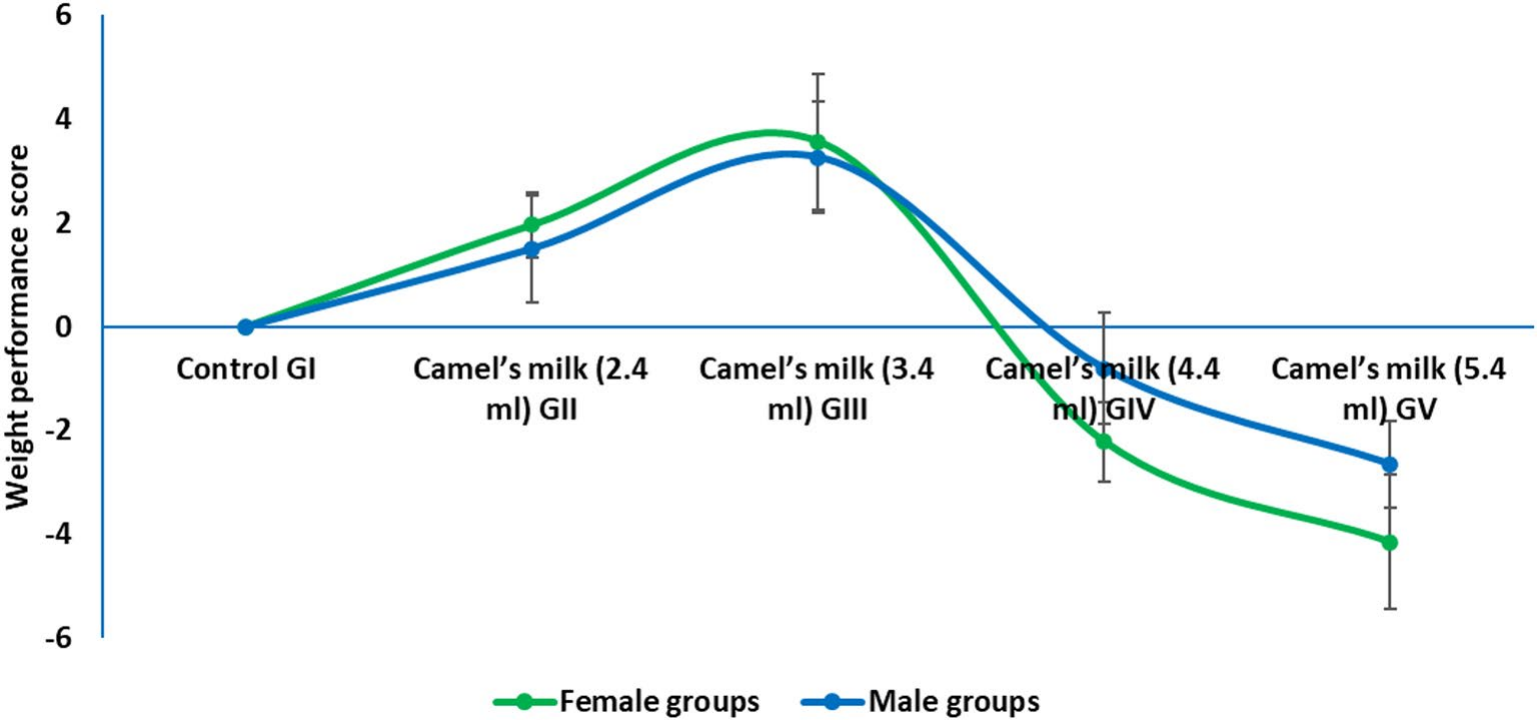
Composite growth performance score in weaning rats administered camel milk for six weeks. The score integrated Z-scores for final body weight and total weight gain, calculated relative to the sex-matched control group (GI).

To provide an integrated assessment of the overall growth response, a composite growth performance Z-score was calculated for each animal by averaging the Z-scores for final body weight and total weight gain, standardized to the sex-matched control group (Fig. 2). In female rats, the score was significantly elevated in the GII (2.4 mL) and GIII (3.4 mL) groups, with the latter showing the most pronounced beneficial effect (Score = 3.56). Conversely, the score was significantly suppressed in the GIV (4.4 mL) and GV (5.4 mL) groups, indicating substantial growth impairment at high doses. A similar pattern was observed in male rats, with the GIII group demonstrating the most robust growth enhancement (Score = 3.26). While the GIV group showed a negative trend, the score was only significantly suppressed in the GV group. These results quantitatively confirmed that the WHO-equivalent dose (GIII) is optimal for promoting growth, while supra-physiological doses exerted a significant suppressive effect, with female rats exhibiting greater susceptibility to high-dose growth impairment.

### Biochemical analysis

**Fig. 3:**
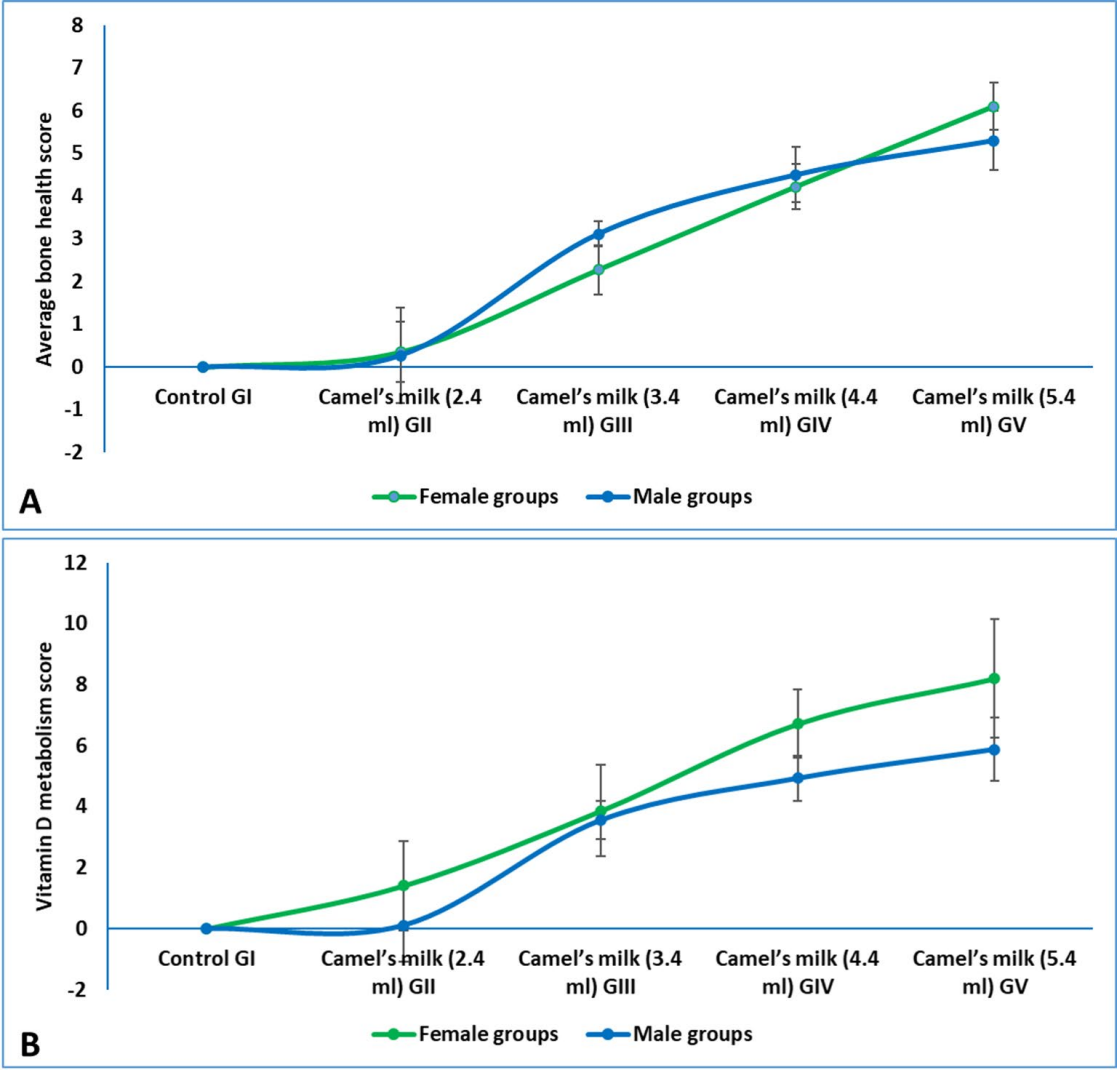
(**A**) average bone health score in weaning rats administered camel milk for six weeks. The score integrated Z-scores for serum total calcium, phosphorus, and calcitriol, calculated relative to the sex-matched control group (GI), (**B**) vitamin D metabolism score in weaning rats administered camel milk for six weeks. The score integrated Z-scores for serum calcifediol and calcitriol, calculated relative to the sex-matched control group (GI).

To provide a clear and integrated summary of the dose-response effects, the results for biochemical parameters were presented as composite Z-scores. The detailed data for individual parameters were available in supplementary materials (figures S1). The composite bone health score, which integrated key parameters of bone metabolism, demonstrated a clear dose-dependent response to camel milk administration in both female and male rats (Fig. 3A). While the score for the low-dose group (GII, 2.4 mL) remained near baseline levels, a significant elevation was observed in the group receiving the WHO-equivalent dose (GIII, 3.4 mL). This positive trend continued in the higher-dose groups (GIV, 4.4 mL and GV, 5.4 mL), which exhibited the highest bone health scores. Furthermore, female rats consistently displayed a more pronounced response at high dose (GV, 5.4 mL) compared to their male counterparts, indicating a sex-specific enhancement of bone health parameters following camel milk consumption. The Vitamin D metabolism score, reflecting the combined levels of calcifediol and calcitriol, also increased in a dose-dependent manner following camel milk administration (Fig. 3B). Like the bone health parameters, the most substantial rise was observed in the highest dose groups (GIV and GV). This trend was more pronounced in female rats, which exhibited significantly higher scores than males at the 4.4 mL and 5.4 mL doses. The strong positive correlation between the Vitamin D metabolism score and the bone health score suggested that the enhancement in bone metabolism was likely mediated, at least in part, by the improved vitamin D status facilitated by camel milk consumption.

### Liver function enzymes

**Fig. 4:**
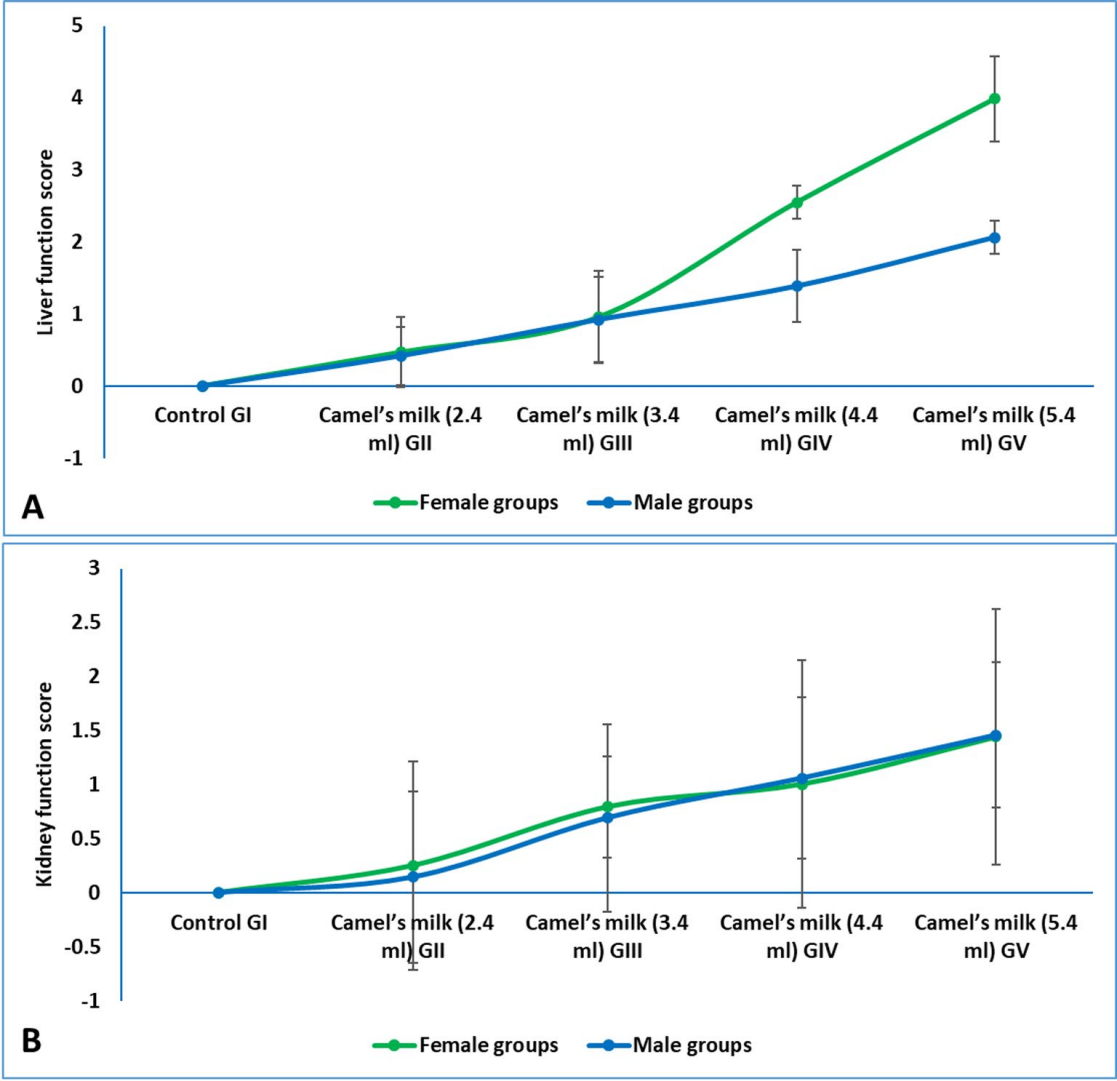
(**A**) liver function score in weaning rats administered camel milk for six weeks. The score integrated Z-scores for serum ALT and AST, calculated relative to the sex-matched control group (GI), (**B**) kidney function score in weaning rats administered camel milk for six weeks. The score integrated Z-scores for urea and creatinine, calculated relative to the sex-matched control group (GI).

The detailed data for individual parameters was available in supplementary materials (figures S2). The composite liver function score, which integrated serum levels of ALT and AST, demonstrated a dose-dependent increase, indicating the elevation of hepatic stress with higher camel milk intake. In female rats, the score remained stable at the 2.4 mL (GII, 0.46) and 3.4 mL (GIII, 0.95) doses but rose markedly at the 4.4 mL (GIV, 2.55) and 5.4 mL (GV, 3.98) doses. A similar pattern was observed in males, with scores of 0.41 (GII) and 0.92 (GIII) escalating to 1.38 (GIV) and 2.06 (GV). This indicated that while the lower and recommended doses had minimal impact on liver enzymes, the supra-physiological doses (4.4 mL and 5.4 mL) induced significant hepatic stress in both sexes, with a more pronounced effect in females (Fig. 4A).

### Kidney function parameters

The detailed data for individual parameters was available in supplementary materials (figures S2). The composite kidney function score, derived from serum urea and creatinine levels, revealed a dose-dependent effect on renal parameters (Fig. 4B). Administration of the low dose of camel milk (2.4 mL, GII) resulted in minimal change to the kidney function score in both female (0.25) and male (0.14) rats compared to the control group. However, a substantial increase was observed beginning with the WHO-equivalent dose (3.4 mL, GIII), where scores rose to 0.79 in females and 0.69 in males. This trend continued in a dose-dependent manner, with the highest scores recorded at the 5.4 mL dose (GV) for both females (1.44) and males (1.46). The results indicated that camel milk intake at and above the 3.4 mL dose places a significant metabolic load on renal function, with a similar magnitude of effect observed in both sexes at the highest dosage.

### Lipid profile

**Fig. 5:**
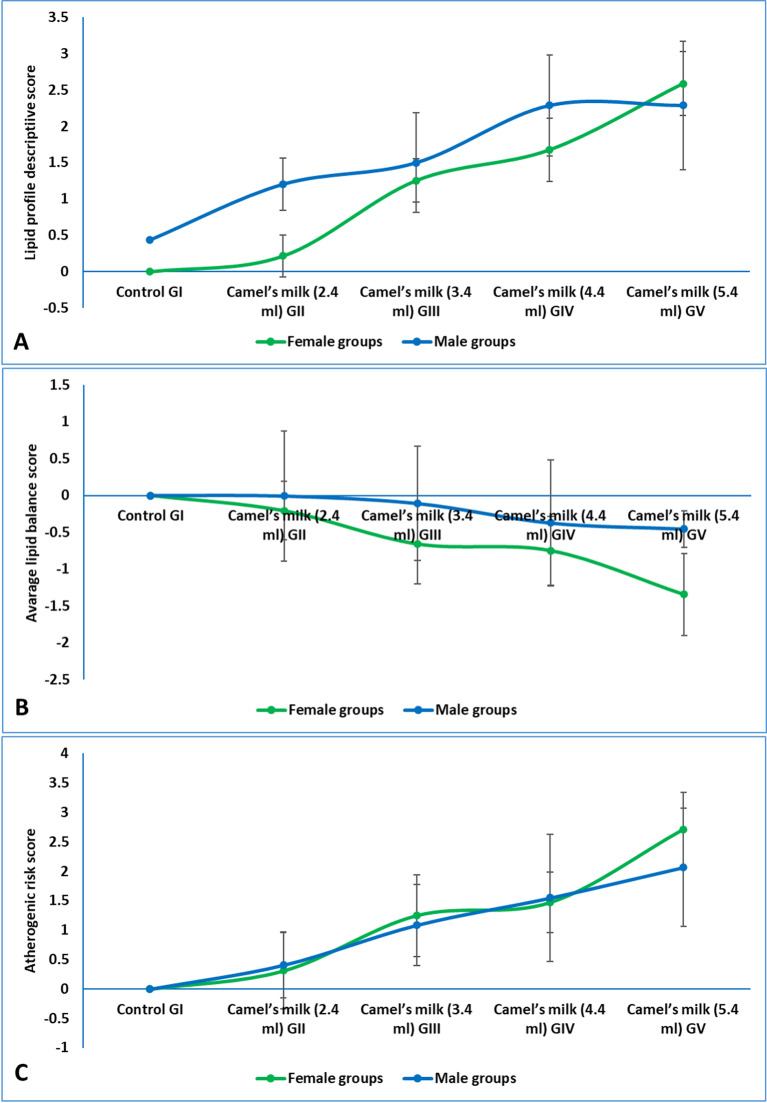
(**A**) lipid profile descriptive score in weaning rats administered camel milk for six weeks. The score integrated Z-scores for serum cholesterol, triglycerides, HDL and LDL, calculated relative to the sex-matched control group (GI), (**B**) average lipid balance score in weaning rats administered camel milk for six weeks. The score integrated Z-scores for triglycerides, HDL and LDL, calculated relative to the sex-matched control group (GI), (**C**) atherogenic risk score in weaning rats administered camel milk for six weeks. The score integrated Z-scores for serum triglycerides and LDL, calculated relative to the sex-matched control group (GI).

To provide a clear and integrated summary of the dose-response effects, the results for lipid profile were presented as composite Z-scores. The detailed data for individual parameters was available in supplementary materials (figures S3). The simple descriptive score for the lipid profile, which integrated total cholesterol, triglycerides, HDL-C, and LDL-C, demonstrated a pronounced dose-dependent elevation in overall serum lipid levels. The score increased marginally in the low-dose group (GII, 2.4 mL) for both females (0.21) and males (1.20). A substantial rise was observed at the WHO-equivalent dose (GIII, 3.4 mL), with scores reaching 1.25 in females and 1.49 in males. This upward trend continued progressively at the higher doses, culminating in the most significant elevation at the 5.4 mL dose (GV), where scores peaked at 2.59 for females and 2.29 for males. The results indicated that camel milk consumption induced a general hyperlipidemic effect, characterized by a concurrent increase in all major lipid fractions, with a consistently stronger effect observed in female rats across all treatment groups (Fig. 5A).

The lipid balance score, which weighs atherogenic lipids (LDL-C, triglycerides) negatively and atheroprotective HDL-C positively, revealed a significant dose-dependent deterioration in lipid profile health. While the low dose (GII, 2.4 mL) resulted in a minimal change for males (−0.007) and a slight negative shift for females (−0.21), the WHO-equivalent dose (GIII, 3.4 mL) induced a more pronounced unfavorable shift in the lipid balance, particularly in females (−0.65). This negative trend intensified dramatically at the highest doses, with the most atherogenic profile observed in the 5.4 mL group (GV) for both females (−1.34) and males (−0.45). The results demonstrated that while lower doses have a modest impact, supra-physiological camel milk intake significantly shifted the lipid profile towards a more atherogenic state, with female rats exhibiting a markedly greater susceptibility to this adverse effect (Fig. 5B).

The atherogenic risk score, calculated from the key risk factors LDL-C and triglycerides, demonstrated a significant and dose-dependent increase in lipid-associated cardiovascular risk (Fig. 5C). The score rose markedly even at the WHO-equivalent dose (GIII, 3.4 mL), increasing to 1.24 in females and 1.08 in males compared to the control baseline. This trend intensified substantially with higher intake, culminating in the greatest risk score at the 5.4 mL dose (GV) for both females (2.71) and males (2.06). The results indicated that camel milk consumption, particularly at doses above the recommended level, significantly elevated the atherogenic risk. Furthermore, while the absolute risk score was higher in males at most doses, the proportional increase from the control baseline was more pronounced in females.

### Immunological parameters

**Fig. 6:**
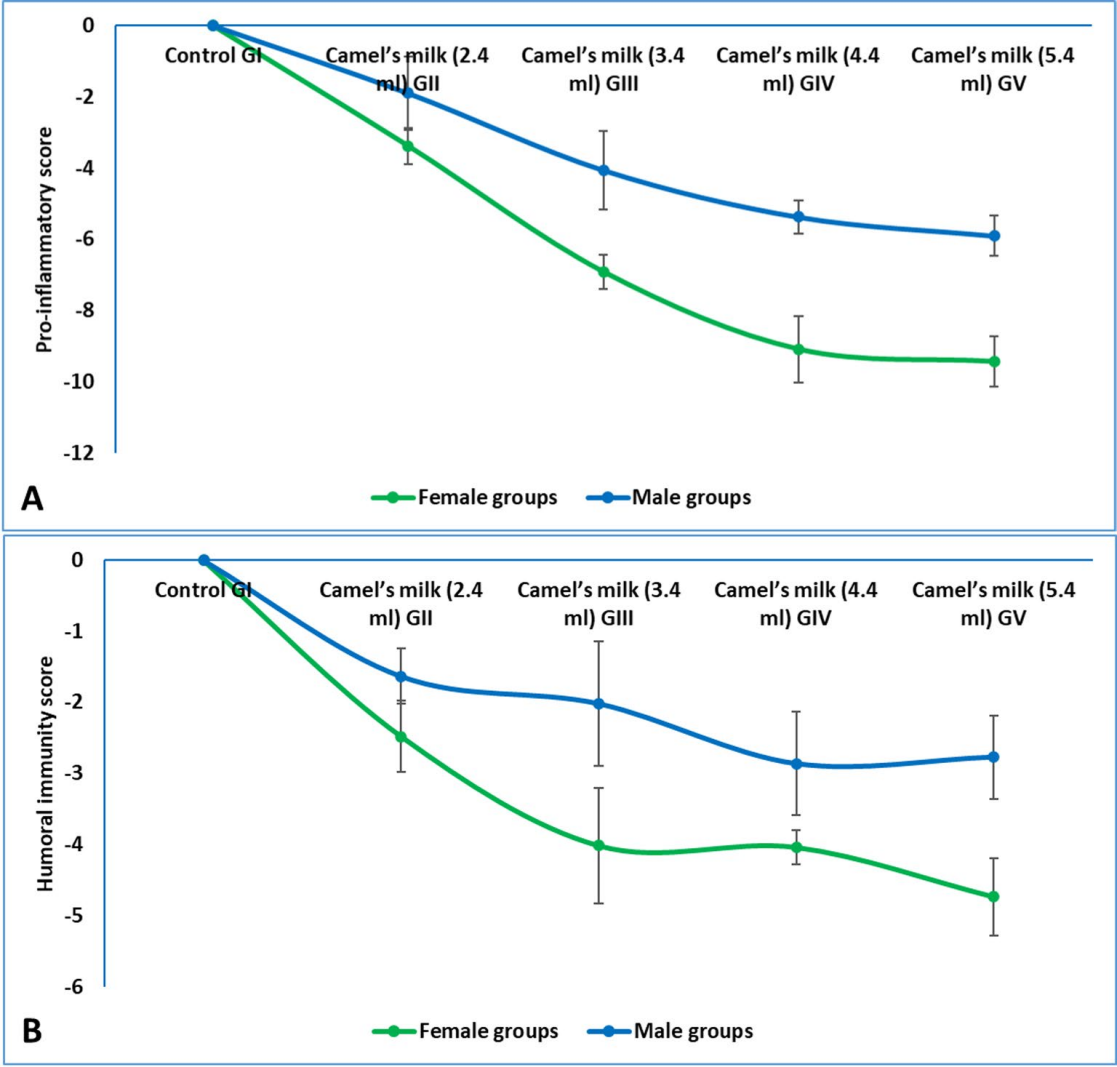
(**A**) Pro-inflammatory descriptive score after immunization with SRBCs in weaning rats administered camel milk. The score integrated Z-scores for key pro-inflammatory cytokines (IL-1β, IL-6 and IL-17), calculated relative to the sex-matched control group (GI), (**B**) Immunoglobulin descriptive score after immunization with SRBCs in weaning rats administered camel milk. The score integrated Z-scores for serum immunoglobulins (IgG and IgA), calculated relative to the sex-matched control group (GI).

The detailed data for individual parameters was available in supplementary materials (figures S4 and table S1). The composite pro-inflammatory cytokine score, derived from serum levels of IL-1β, IL-6, and IL-17 after SRBCs immunization, revealed a powerful and dose-dependent anti-inflammatory effect of camel milk in both sexes (Fig. 6A). Female rats exhibited a profound suppression of the score, which decreased dramatically from the 2.4 mL dose (−3.38) to the 5.4 mL dose (−9.43), indicating a very strong, progressive dampening of the inflammatory response. A similar, significant dose-dependent suppression was observed in male rats, with scores declining from − 1.89 at the 2.4 mL dose to −5.89 at the 5.4 mL dose. Crucially, the magnitude of suppression was substantially greater in females than in males at every corresponding dose level, demonstrating a marked sexual dimorphism in the anti-inflammatory potency of camel milk. These findings conclusively showed that camel milk administration potently attenuated the pro-inflammatory cytokine cascade following an immune challenge, with a consistently more robust effect in female weaning rats.

The composite humoral immunity score, which integrated the serum levels of IgM and IgG after immunization, demonstrated a significant and dose-dependent suppressive effect of camel milk on the overall antibody response in both sexes (Fig. 6B). In female rats, the score was significantly suppressed at all administered doses, showing a progressive decline from the 2.4 mL dose (−2.47) to the 5.4 mL dose (−4.73). Male rats also exhibited a significant suppression of the humoral score across all treatment groups, with the most pronounced effect observed at the 4.4 mL dose (−2.86). Notably, the magnitude of suppression was consistently greater in females than in males across all corresponding dose groups, highlighting a clear sexual dimorphism in the susceptibility of the antibody-mediated immune response to camel milk modulation. These results confirmed that camel milk intake effectively attenuated the humoral immune response to SRBCs in a dose-responsive manner, with a more potent effect observed in female weaning rats.

### Dose-response effects of camel milk

The comprehensive dose-response analysis revealed that camel milk exerted distinct, sex-specific effects across physiological systems, with a clear exchange between benefits and risks at higher doses (Table 1). Bone health and vitamin D metabolism were consistently and strongly enhanced in both sexes, showing significant, large-to-very large improvements (Cohen’s d > 2.0) at the WHO-equivalent dose (3.4 mL) and above. However, significant adverse effects emerged at higher intakes. Hepatic stress was observed in both sexes at the 4.4 mL and 5.4 mL doses, evidenced by significant elevations in liver enzymes, with a more pronounced effect in females. A notable sex difference was identified in renal response; while female kidney parameters remained unaffected, males exhibited a significant increase in serum creatinine at the highest dose (5.4 mL). The lipid profile showed an early atherogenic shift, with significant increases in triglycerides from the 3.4 mL dose in both sexes, though this was partially balanced by a beneficial rise in HDL-C in males at the highest doses. Collectively, these findings establish the 3.4 mL dose as optimal, providing substantial benefits to bone metabolism without inducing significant hepatic, renal, or lipid stress. The detailed statistical results for all parameters, including mean differences, 95% confidence intervals, and Cohen’s d values, were provided in supplementary tables S5 (female) and S6 (male).

The comprehensive immunological profile revealed that camel milk acted as a potent, dose-dependent immunomodulator, with effects exhibiting significant sexual dimorphism (Table 2). A profound anti-inflammatory effect was observed in both sexes, characterized by the significant suppression of key pro-inflammatory cytokines (IL-1β, IL-6, IL-17, IL-18), often with very large effect sizes (e.g., female IL-17, Cohen’s d = −11.62). This suppressive effect was more consistent and potent in females, where it was significant at all doses, while males typically exhibited a threshold effect starting at the 3.4 mL dose. The humoral immune response was differentially regulated. The early IgM response was strongly suppressed in both females and males at all doses. In contrast, the adaptive IgG response was significantly suppressed only in females at medium and high doses, whereas it remained completely unaffected in males at any dose level. These findings collectively demonstrated that camel milk possessed strong anti-inflammatory properties and can regulate antibody production, but its effects were critically dependent on both the administered dose and the sex of the animal, with female rats exhibiting a broader and more sensitive immunomodulatory response. The detailed statistical results for all cytokines/immunoglobulin, including mean differences, 95% confidence intervals, and Cohen’s d values, were provided in supplementary tables S7 (female) and S8 (male).

**Table 1:** Summary of the dose-dependent effects of camel milk on key physiological systems in weaning rats.

Physiological system	Sex	Key finding	Optimal dose (3.4 mL) Effect	High-dose (5.4 mL) Risk
Bone & vitamin D	Female	Strong, dose-dependent benefit	Large Improvement	Continuous Benefit
	Male	Strong, dose-dependent benefit	Large Improvement	Continuous Benefit
Liver function	Female	Significant stress at high doses	No Effect	Strong Adverse Effect
	Male	Significant stress at high doses	No Effect	Adverse Effect
Kidney function	Female	No significant impact	No Effect	No Effect
	Male	Elevated creatinine at high doses	No Effect	Adverse Effect
Lipid profile	Female	Atherogenic shift at high doses	Early Adverse Effect	Strong Adverse Effect
	Male	Mixed response (increase TG and HDL)	Early Adverse Effect	Adverse Effect

**Table 2:** Summary of the dose-dependent Immunomodulatory effects of camel milk in weaning rats after SRBCs immunization.

Physiological system	Parameter	Sex	Key finding	Effective dose(s)	Magnitude (Max Cohen’s d)
Pro-inflammatory cytokines	IL-1β, IL-6, IL-17, IL-18	Female	Potent and consistent suppression of the entire pro-inflammatory cascade.	All Doses (GII-GV)	−11.62 (IL-17, GV)
	IL-1β, IL-6, IL-17, IL-18	Male	Significant suppression, with a clear threshold effect for most cytokines.	GIII, GIV, GV (IL-18: All Doses)	−6.74 (IL-1β, GV)
Humoral immunity	IgM	Female	Strong and progressive suppression of the early antibody response.	All Doses (GII-GV)	−8.39 (GV)
	IgM	Male	Potent suppression of the early antibody response.	All Doses (GII-GV)	−5.85 (GV)
	IgG	Female	Significant suppression at medium and high doses.	GIII, GIV, GV	−2.27 (GV)
	IgG	Male	No significant effect at any dose.	None	Not Significant

### Histopathological results

To confirm the biochemical findings at the tissue level, a histopathological analysis was performed on the spleen, liver, and kidneys. The results strongly supported the dose-dependent effects observed in our biochemical assays. In the spleen, high-dose camel milk administration (GIV and GV) was associated with morphological signs of immune activation and stress, including marked sinusoidal congestion and the presence of apoptotic lymphocytes. In the liver, a clear dose-dependent progression of injury was evident. While the control, low-dose (GII), and optimal-dose (GIII) groups showed normal hepatic architecture, the high-dose groups (GIV and GV) exhibited significant pathology, including portal inflammation, vascular congestion, and extensive hepatocyte apoptosis, directly corroborating the observed elevations in serum ALT and AST. Examination of the kidneys revealed preserved tissue architecture across all groups. Only minimal, scattered apoptosis was noted, with no evidence of overt tubular necrosis or glomerular damage. This indicated that the elevated serum creatinine levels, particularly in high-dose male group, were likely indicative of a functional renal load rather than irreversible structural injury. Representative photomicrographs were provided in supplementary materials (figures S5, S6 and S7).

## Discussion

Research findings demonstrated that camel milk possesses distinct advantages over other milk types, containing nutrients^36^ with documented antibacterial, antioxidant^37^, and angiotensin-converting enzyme (ACE)-inhibitory properties that may protect against cardiovascular diseases^38,39^. Camel milk consumption supported cardiovascular health and showed potential for mitigating diabetic symptoms^40^. While the immunomodulatory and metabolic benefits of camel milk have been previously established^12,22,24^, the critical question of dose-dependent efficacy and safety remains largely unexplored. Prior studies have primarily utilized single doses, limiting their ability to define optimal intake levels or identify potential toxicity thresholds. Our study fundamentally advances the field by systematically mapping a complete dose-response curve for camel milk in a pediatric model. This approach allows us to move beyond confirming its bioactivity to providing crucial translational insights into the balance between its health benefits and metabolic risks, thereby addressing a significant gap in the literature on camel milk application in nutrition.

Compositional analysis revealed that camel milk shares remarkable similarities with human milk, particularly when compared to donkey and mare milk, a feature likely contributing to its health-promoting properties^41^. However, it differs substantially from other ruminant milk in key aspects^42^, notably containing more ash but far less lactose than goat, cow, and bovine milk^24^. Our analysis confirmed camel milk contained proteins (2.4%), fats (2.9%), and lactose (2.6%), with high water content (92.1%), aligning with reported protein ranges of 2.1–4.9%^43^. The lactose content was consistent with previous observations^37^ and comparable to goat milk^44^. Mineral analysis, however, revealed substantially higher levels of calcium (1209.0 mg/100 mL), phosphorus (934.0 mg/100 mL), and potassium (1707.0 mg/100 mL) than those previously documented^45^, alongside significant sodium (447.0 mg/100 mL). A comprehensive profile of both water-soluble (vitamin C, B complex) and fat-soluble vitamins (A, E, D3) was also identified.

Lipid profiling provided new insights, confirming and extending findings of diverse lipid subclasses and major groups^46^. The remarkable similarity between camel and human milk lipid profiles (87 distinct lipids) compared to the greater differences with cow (903 lipids) and goat milk (918 lipids) underscores its potential as a superior nutritional alternative^47^. A detailed breakdown (90.08% triglycerides, 4.28% phosphatidylcholine, 3.11% sphingomyelin) mechanistically supports the proposal that camel milk’s phospholipids could improve infant formula^48^. The amino acid profile confirmed camel milk as a complete protein source, with essential amino acids (34.3 g/100 g total) exceeding FAO/WHO recommendations^49^. Glutamic acid was most abundant (19.1 g/100 g), followed by proline (12.0 g/100 g) and aspartic acid (5.7 g/100 g), with a balanced profile of essential amino acids including leucine (8.2 g/100 g). The phospholipid distribution and fatty acid profile (SFA: 58.4%; MUFA: 35.8%; PUFA: 5.8%) offer functional insights. The SFA fraction was rich in palmitic acid (27.9%), stearic acid (10.3%), myristic acid (7.4%), and lauric acid (6.1%). The MUFA profile was dominated by oleic acid (31.3%). Although total PUFA content is lower than in human milk fat^50^, its unique sn-2 positioning- characterized by reduced SFA, elevated MUFA, and favorable phospholipid distributions- enhances fat absorption and lipid metabolism^51^. This distinctive lipid architecture, aligning with and extending previous work^48,50^, may explain camel milk’s anti-inflammatory and cardiovascular protective effects.

The dose selection in this study was carefully calibrated based on established conversion factors from previous research by Amr and Farid^12^ and Amr et al.^22^, where the administration of 3.4 mL of milk to 3-week-old rats was determined to be equivalent to the WHO-recommended daily serving of 473.1 mL (approximately 2 cups) for 19-month-old human infants. Using this validated conversion criteria, the study examined a range of doses in a rat model: 2.4, 3.4, 4.4, and 5.4 mL. These doses were strategically selected to bracket the USDA/Center for Nutrition Policy and Promotion (CNPP) recommendations of 393–473.1.1 mL (1⅔−2 cups) per day for toddlers aged 12–24 months^52^, allowing to systematically evaluate the effects of suboptimal, optimal, and supraoptimal camel milk intake on health parameters in both male and female Sprague Dawley rats across five experimental groups (GI: control; GII: 2.4 mL; GIII: 3.4 mL; GIV: 4.4 mL; GV: 5.4 mL).

Given the critical importance of bone health during early development and the prevalence of childhood disorders like rickets, stunting, and low bone mass stemming from deficiencies in calcium, phosphorus, or vitamin D^53^, this study conducted a comprehensive assessment of bone metabolism. We measured serum levels of total calcium, ionized calcium (the physiologically active fraction), phosphorus, calcifediol (25-OH vitamin D3, the storage form), and calcitriol (1,25(OH)2 vitamin D3, the active hormonal form). The results demonstrated a clear dose-response relationship. Female rats consistently exhibited higher calcium levels than males, a sexual dimorphism that may be explained by the established role of estradiol (E2) in calcium homeostasis. In previous research, E2 has been shown to enhance intestinal calcium absorption and stimulate renal 1-α hydroxylase activity to promote calcitriol synthesis^54^. This well-documented mechanism provides a plausible explanation for our findings, particularly given the significantly higher physiological reference ranges for E2 in young females compared to males^55^. While circulating hormone levels were not quantified in our study, this robust physiological mechanism provides a plausible explanation for our results. Dose-dependent increases in phosphorus and vitamin D metabolites were also observed, reflecting camel milk’s high native content of phosphorus (934 mg/kg) and vitamin D3 (3.1 mg/kg). This follows the canonical metabolic pathway where dietary vitamin D is converted to calcifediol in the liver and then to bioactive calcitriol in the kidney^22^, creating a robust system for supporting bone mineralization.

Our findings revealed distinct dose-dependent effects. The 2.4 mL dose (GII) was subtherapeutic, producing no significant elevation in any bone marker compared to controls. This intake level (334 mL human equivalent) falls substantially below both the WHO recommendation (473.1 mL) and the minimum USDA CNPP guideline (393 mL)^52^, failing to meet the mineral demands for rapid skeletal growth. In striking contrast, the WHO-equivalent 3.4 mL dose (GIII) significantly elevated all measured bone parameters. This positive effect was progressively enhanced at the 4.4 mL (GIV) and 5.4 mL (GV) doses, establishing a clear dose-response relationship. These results underscore that camel milk’s rich mineral and vitamin D3 content can effectively support pediatric bone development, but only when administered at adequate doses.

The hepatic and renal systems are crucial for metabolic homeostasis and detoxification. The liver, as the primary metabolic center, orchestrates the metabolism of proteins, carbohydrates, and lipids while performing critical detoxification functions^21^. Its health is monitored via key enzymes, ALT and AST. Although present in various tissues, elevated serum levels of these pyridoxal phosphate (PLP)-dependent transaminases are sensitive biomarkers of hepatic cellular damage^56^. Renal function is primarily assessed through blood urea, the end-product of protein catabolism; and serum creatinine, a byproduct of muscle metabolism. Elevated levels of these markers indicate impaired kidney function and reduced glomerular filtration rate, respectively^57^. The evaluation of camel milk’s dose-dependent effects revealed critical thresholds for hepatic and renal tolerance. Doses of 2.4 mL (GII) and 3.4 mL (GIII) maintained ALT and AST within normal physiological ranges in both sexes, indicating preserved liver integrity. In contrast, the 4.4 mL (GIV) and 5.4 mL (GV) doses induced significant enzyme elevations, suggesting hepatic stress. This dose-response pattern was mirrored in renal parameters, where higher doses caused marked elevations in urea and creatinine, particularly in males, a finding attributable to their greater muscle mass and consequent higher protein turnover^58^.

The underlying pathophysiology involves the metabolic burden from camel milk’s rich protein (2.4%) and amino acid content (34.3 g/100 g essential; 52.3 g/100 g non-essential). At supraphysiological doses, the liver’s metabolic capacity is overwhelmed, triggering accelerated transamination (via ALT/AST). Subsequent deamination is expected to generate ammonia, leading to compensatory urea cycle activation and secondary renal stress from increased nitrogenous waste excretion^59^. While our study did not measure ammonia directly, this established metabolic cascade provides a plausible pathway for the observed hepatic and renal biomarker elevations. The potential for ammonia accumulation also suggests a theoretical risk for neurological sequelae, which warrants investigation in future studies specifically designed to assess neurotoxicity.

Lipid profile analysis, a panel of blood tests used to identify dyslipidemia and associated disease risks^60^, provided valuable insights into the metabolic effects of camel milk. Female rats consistently exhibited higher baseline lipid levels than males across all groups, consistent with established physiological sexual dimorphism attributed to hormonal influences and differences in body composition^61^. A clear dose-dependent response was observed. The highest dose (GV, 5.4 mL) significantly elevated total cholesterol, triglycerides, and HDL-c in both sexes, while LDL-c increased significantly only in females. The intermediate dose (GIV, 4.4 mL) induced similar but less pronounced changes. Notably, the WHO-equivalent dose (GIII, 3.4 mL) selectively elevated triglycerides while maintaining other lipid fractions, and the lowest dose (GII, 2.4 mL) showed no significant alterations. These findings can be explained by camel milk’s unique lipid composition, which includes beneficial monounsaturated (35.8%) and polyunsaturated (5.8%) fatty acids, alongside a moderate content of predominantly long-chain saturated fats (58.4%)^12,22^. The results indicate a threshold effect, where doses at or below the WHO recommendation maintain favorable lipid profiles, while higher doses induce a general hyperlipidemic state, potentially increasing the risk of lipid-related complications. The selective triglyceride response at the 3.4 mL dose suggests distinct metabolic handling for different lipid fractions. These results support camel milk’s role as a functional food for metabolic health at appropriate doses and underscore the critical importance of considering sex-specific responses in nutritional interventions.

The immunomodulatory effects of camel milk were systematically assessed using SRBCs as a T-dependent antigen, enabling comprehensive monitoring of the immune cascade from antigen presentation to antibody production. Female rats exhibited consistently stronger immune responses than males, with significantly higher levels of pro-inflammatory cytokines (IL-1β, IL-6, IL-17) and immunoglobulins (IgM, IgG), a phenomenon likely attributable to their greater adiposity and associated inflammatory adipokine production that enhances macrophage recruitment and activation^62^. It is important to note that while this provides a strong mechanistic framework for our findings, the specific contributions of hormones and adipokines were not directly measured in this study. Baseline measurements confirmed comparable immune parameters across all groups prior to immunization. The antibody response profile revealed that while immunization effectively stimulated IgM production, IgG responses were consistently more robust in females across all groups. Notably, all camel milk-fed groups showed reduced immunoglobulin levels relative to controls, suggesting a potential regulatory effect on antibody production.

The consistently more robust immune activation observed in female rats is a well-documented manifestation of sexual dimorphism in immunity. This heightened immunoreactivity in females is fundamentally driven by the immunomodulatory effects of sex hormones, particularly 17β-oestradiol (E2)^63^. E2 enhances immune function by modulating the expression of a wide array of genes through the activation of estrogen receptors ERα and Erβ^64,65^, leading to stronger innate and adaptive immune responses^66^. Specifically, E2 potentiates the humoral arm of immunity by enhancing B cell development, antibody production^67^, and upregulating activation-induced cytidine deaminase (AID), which is essential for antibody class-switch recombination and affinity maturation, thereby directly contributing to higher antibody titers^68,69^. Concurrently, E2 promotes a heightened pro-inflammatory state by enhancing the production of pro-inflammatory cytokines^70^ and increasing the expression of Toll-like receptors (TLRs), which are crucial for initiating inflammatory responses^71,72^. This potent hormonal influence is further compounded by the typically greater adiposity in females, which contributes to a heightened inflammatory milieu through increased production of adipokines^62,73^. In summary, this enhanced female immune phenotype is the result of the combined effects of genetics^74^, epigenetics, and sex steroid hormones, which is beneficial for pathogen clearance but also increases susceptibility to inflammatory and autoimmune diseases^75^. This provides a compelling multi-faceted physiological rationale for the more robust immune activation observed in female rats in our study following immunization and camel milk administration.

Camel milk’s unique nutritional composition can mechanistically explain these immunological effects. The milk contains potent antioxidant components, including methionine (1.9 g/100 g), which generates immunomodulatory homocysteine that functions as an NO inhibitor^76^, and vitamin C, which enhances neutrophil chemotaxis and microbial clearance^77^. The lipid fraction demonstrates significant immunoregulatory properties through its MUFA (31.3% oleic acid) and PUFA (2.7% α-linolenic acid, 3.1% linoleic acid) that modulate dendritic cell function^78^, along with SFA (palmitic acid 27.9%, stearic acid 10.3%) that promote dendritic cells maturation and T-cell activation. Furthermore, the substantial phospholipid content, including glycerophospholipids (phosphatidyl ethanolamine, 4.7 mg/100 mL) and sphingomyelins (3.2 mg/100 mL), contributes to immune regulation by influencing innate immune cell activities and mediating cellular signaling pathways involved in hemostasis and vascular inflammation^79,81,81^.

The histopathological analysis provided crucial tissue-level confirmation of the biochemical results. The significant elevations in serum ALT and AST observed in the high-dose groups (GIV and GV) were directly corroborated by hepatic tissue damage, evidenced histologically by moderate portal inflammation, marked vascular congestion, and extensive hepatocyte apoptosis. Similarly, the absence of significant histological damage in the kidneys, which showed preserved architecture with only minimal apoptosis, despite elevated serum creatinine, particularly in males, indicates that the renal impact was primarily a functional stress rather than overt structural injury. Furthermore, the splenic findings of sinusoidal congestion and apoptotic lymphocytes in high-dose groups aligned with the potent immunomodulatory effects seen in serum, suggesting that the systemic anti-inflammatory state may be linked to increased immune cell turnover within this primary lymphoid organ. This strong concordance between biochemical markers and tissue morphology reinforces the dose-dependent hepato-renal stress and immunomodulation induced by supra-physiological camel milk intake.

Our study revealed a significant, multi-compartmental immunomodulatory effect of high-dose camel milk. While serum profiling demonstrated a profound systemic anti-inflammatory state, characterized by significant suppression of key pro-inflammatory cytokines, histopathological analysis of the spleen presented a more complex picture. The observed marked sinusoidal congestion and scattered apoptotic lymphocytes in the high-dose groups suggest a state of significant immune activity and stress within this lymphoid organ. This apparent conflict can be explained by considering the distinct roles of systemic signaling versus tissue-level processing. We hypothesize that the potent bioactives in camel milk may dampen systemic inflammation, while simultaneously imposing a substantial metabolic and immunological load on the spleen as the primary site for antigen clearance and immune cell turnover^82,83^. The observed apoptosis could represent a potential cellular mechanism contributing to the overall systemic immunosuppression, a phenomenon that has been documented for other bioactive immunomodulators^84^. This highlights that the immunomodulatory action of camel milk is not monolithic but involves distinct, and potentially opposing, effects in different physiological compartments.

A pivotal and novel finding of this dose-response investigation is the identification of a clear therapeutic window for camel milk intake. We demonstrate that the health benefits of camel milk are not linear but instead follow a U-shaped curve. While supra-physiological doses (4.4 and 5.4 mL) enhanced certain immune parameters, this occurred at the cost of significant hepatic and renal stress, indicating a trade-off between immunomodulation and metabolic toxicity. This finding is critical for public health guidance, as it cautions against the assumption that ‘if some are good, more is better.’ In contrast, the WHO-equivalent dose (3.4 mL) provided significant enhancements in immune function and bone health without any detectable adverse effects, establishing it as the optimal dose for balancing efficacy and safety in a weaning model.

However, several limitations have to be acknowledged, including the inherent constraints of animal models in predicting human physiological responses. While the 6-week duration was sufficient to model the critical early post-weaning period and capture significant changes in immune and metabolic parameters, it is a study limitation that it cannot assess the long-term or lifelong developmental outcomes of camel milk consumption. Furthermore, while our sample size (*n* = 10 per group) is standard and provided robust statistical power for the large effect sizes expected in this nutritional model, future studies with even larger cohorts could be designed to detect more subtle effects. Additionally, the single-timepoint immune assessment represents an opportunity for methodological refinement in future studies. Another important consideration is the potential variability in camel milk composition, which can be influenced by factors such as lactation stage, breed, diet, and geography. To ensure consistency and reproducibility in our intervention, the milk used in this study was sourced from a controlled herd and pooled from multiple animals within a specific, stable lactation period (4th-9th week post-delivery). While this approach minimizes batch-to-batch variation within the study, it is a limitation that our findings are tied to the specific compositional profile reported herein. Furthermore, while the observed sexual dimorphism in metabolic and immune parameters aligns with established literature, the study did not measure sex hormone levels or specific adipokine profiles. Therefore, the precise endocrine and molecular mechanisms underlying these sex-specific differences in our model remain to be fully elucidated in future work. Future studies should investigate how variations in milk composition, particularly in key bioactive components, influence the observed dose-response effects. Such research would be invaluable for standardizing camel milk-based nutritional products. Also, we recommend the analysis of gut microbiota, which would be crucial to fully understand the prebiotic properties of camel milk and its role in the observed metabolic and immunological effects.

In conclusion, our comprehensive dose-response analysis provides a significant advance over previous single-dose studies by defining the precise conditions under which camel milk exerts its greatest benefit. The study established that a dose of 3.4 mL for rats (473 mL human equivalent) optimally enhances immune function and bone metabolism without inducing hepatic or renal stress, while higher doses risk metabolic toxicity. These findings provide essential evidence-based dosing guidelines for the potential use of camel milk as a nutritional supplement during the critical weaning period. Future clinical trials are warranted to translate these precise dosing recommendations into practical applications for human infant nutrition.

## Supplementary Information

Below is the link to the electronic supplementary material.

Supplementary Material 1

## Data Availability

All data are available from the corresponding author on a reasonable request.

## Abbreviations

ACE: Angiotensin-converting enzyme
AI: After immunization
ALT: Alanine aminotransferase
ANOVA: One way analysis of variance
APRI: Animal production research institute
AST: Aspartate aminotransferase
BI: Before immunization
Ca: Calcium
CNPP: Center for nutrition policy and promotion
DC: Dendritic cells
E2: Estradiol
ELISA: Enzyme-linked immunosorbent assay
FAO: Food and agriculture organization
Fe: Iron
GC: Gas chromatography
GPLs: Glycerophospholipids
HCl: Hydrochloric acid
HDL-c: High density lipoprotein cholesterol
HPLC: High performance liquid chromatography
ICP-OES: Inductively coupled plasma-optical emission spectrometry
Ig: Immunoglobulin
IL: Interleukin
K: Potassium
KOH: Potassium hydroxide
LDL-c: Low density lipoprotein cholesterol
MHZ: Mega hertz
MUFA: Monounsaturated fatty acids
Na: Sodium
NMR: Nuclear magnetic resonance
P: Phosphorus
pH: Power of hydrogen
PLP: Pyridoxal phosphate
PUFA: Polyunsaturated fatty acids
RP-HPLC: Reversed phase- high performance liquid chromatography
rpm: Round per minute
SD: Standard deviation
SFA: Saturated fatty acids
SGOT: Serum glutamic oxaloacetic transaminase
SGPT: Serum glutamic pyruvic transaminase
SRBCs: Sheep red blood cells
USDA: United States department of agriculture
UV: Ultra-violet
WHO: World Health Organization
Zn: Zinc
