# Supplementary material for: Dose-dependent effects of camel milk on immune function and metabolic health in weaning rats
Source: Sci Rep. 2026 Feb 3;16:4802. doi: 10.1038/s41598-026-35775-0 (PMC12873125; doi:10.1038/s41598-026-35775-0)
Supplement: Supplementary file 1 — Supplementary Material 1 [file 41598_2026_35775_MOESM1_ESM.pdf]

1. **Biochemical analysis:**

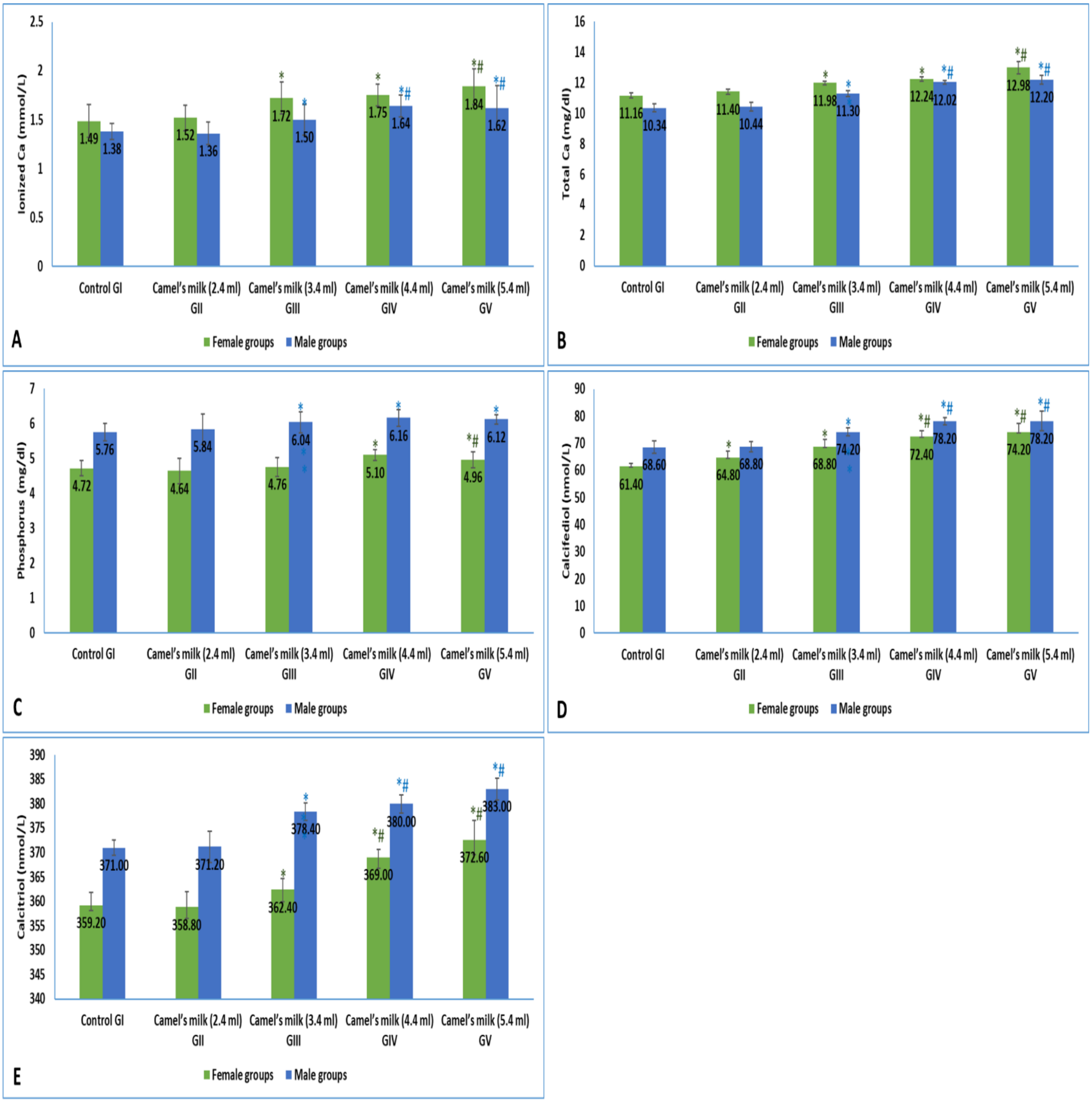

Figure S1: serum levels of ionized Ca (A), total Ca (B), P (C), calcifediol (D) and calcitriol (E) in different female and male animal groups. For female or male groups, results were expressed as mean±SD where * represented the significance (p<0.05) with respect to control GI and # represented the significance (p<0.05) with respect to camel's milk (3.4 mL) GIII.

1.1. Ionized Ca:

Serum level of ionized Ca was higher in female groups than male groups. No significant difference was observed in the ionized Ca level between control GI and camel's milk (2.4 mL) GII either female or male groups (figure S1A). Ionized Ca level increased with increasing the administrated dose of camel milk in female (1.72, 1.75 and 1.84 nmol/L for GIII, GIV and GV, respectively) and male groups (1.50, 1.64 and 1.62 nmol/L for GIII, GIV and GV, respectively).

1.2. Total Ca:

Female groups showed higher levels of total Ca than male groups (figure S1B). For female and male groups, GIII (received 3.4 mL of camel milk) showed a significant elevation in total Ca level (11.98 and 11.30 mg/dl, respectively) when compared to control GI (11.16 and 10.34 mg/dl, respectively). Also, GIV and GV (received 4.4 and 5.4 mL, respectively) showed higher level when compared to GI or GIII (female groups, GIV: 12.24 and GV: 12.98 Vs GI: 11.16 and GIII: 11.98 mg/dl; male groups, GIV: 12.02 and GV: 12.20 Vs GI: 10.34 and GIII: 11.30 mg/dl).

1.3. Phosphorus:

Serum level of P in female groups was lower than those of male groups (figure S1C). Female and male animals received 2.4 mL of camel milk (GII) showed no significant difference in P level when compared to control GI. Female GIII (received 3.4 mL of camel milk) showed P level (4.76 mg/dl) similar to those of GI (4.72 mg/dl) and GII (4.64 mg/dl). On the other hand, male GIII showed significant high P level (6.04 mg/dl) when compared to control GI (5.76 mg/dl). Female GIV (received 4.4 mL camel milk) and GV (received 5.4 mL camel milk) showed high levels (5.10 and 4.96 mg/dl, respectively) when compared to those of GI and GIII. Male GIII, GIV and GV showed an increase in P level in a dose-dependent manner when compared to GI and GII.

1.4. Calcifediol:

Male groups showed high level of calcifediol when compared to female groups (figure S1D). In female groups, calcifediol level increased in a dose-dependent manner (64.80, 68.80, 72.40 and 74.20 nmol/L for GII, GIII, GIV and GV, respectively). For male rats, no significant difference was observed between GI and GII (68.60 and 68.80 nmol/L, respectively). However, a significant increase in calcifediol level was observed for camel milk dose of 3.4 and 4.4 mL which can be observed in male GIII and GIV (74.20 and 78.20 nmol/L). No significant difference was observed in calcifediol level between male GIV and GV.

1.5. Calcitriol:

Male groups showed high level of calcitriol when compared to female groups (figure S1E). In female animals, GIII (received 3.4 mL camel milk) showed a significant increase in the calcitriol level (362.40 nmol/L) when compared to control GI (359.20 nmol/L). Female GIV and GV (received 4.4 and 5.4 mL camel milk, respectively) showed a significant elevation in calcitriol level when compared to their corresponding control GI and GIII (369.00 and 372.6 nmol/L, respectively). The same behavior was observed in male groups.

2. Liver function enzymes:

2.1. ALT:

ALT level was higher in male GIII and GV groups compared to their female counterparts. In female groups, a significant elevation in ALT level was observed in GIV (69.60 U/L) and GV (71.00 U/L) (received 4.4 and 5.4 mL of camel's milk, respectively) when compared to control GI (65.20 U/L) or GIII (66.60 U/L). In male groups, GIII (received 3.4 mL of camel's milk) showed a significantly elevated ALT level (68.20 U/L) when compared to GI (65.40 U/L). Both GIV and GV (69.80 and 72.00 U/L, respectively) showed a significantly elevated ALT level when compared to GI and GIII (65.40 and 68.20 U/L, respectively) (figure S2A).

2.2. AST:

All male groups, except GV, showed higher level of AST than female groups. In both female and male groups, no significant difference was observed among control GI, GII and GIII. Female GIV and GV have a significant high AST level (186.00 and 191.80 U/L, respectively) when compared to those of control GI or GIII (178.40 and 181.60 U/L, respectively. Only male GV (received 5.4 mL of camel's milk) showed a significant high AST level when compared to GIII (received 3.4 mL of camel's milk) (189.60 and 187.00 U/L for GV and GIII, respectively) (figure S2B).

3. Kidney function parameters:

Levels of urea and creatinine were higher in male groups than in female groups.

3.1. Urea:

Administration of 4.4 or 5.4 mL of camel's milk significantly elevated urea level in male and female GIV and GV, respectively, when compared to their corresponding control GI or GIII (received 3.4 mL of camel's milk) (figure S2C). No significant difference was observed in urea level among GI, GII and GIII in female (47.60, 48.00 and 48.20 mg/dl, respectively) or male (48.80, 49.00 and 49.40 mg/dl, respectively) rats.

3.2. Creatinine:

Female and male GIII showed a significant elevation in creatinine level (0.52 and 0.56 mg/dl, respectively) when compared to their corresponding control GI (0.42 and 0.44 mg/dl for female and male rats) (figure S2D). The same behavior in creatinine level was observed with camel's milk dose of 4.4 mL (GIV) in female and male rats. For female GV, no significant difference was observed in creatinine level (0.54 mg/dl) when compared to female GIII (0.52 mg/dl). On the other hand, a significant elevation in creatinine level (0.62 mg/dl) was observed in male GV when compared to male GIII (0.56 mg/dl).

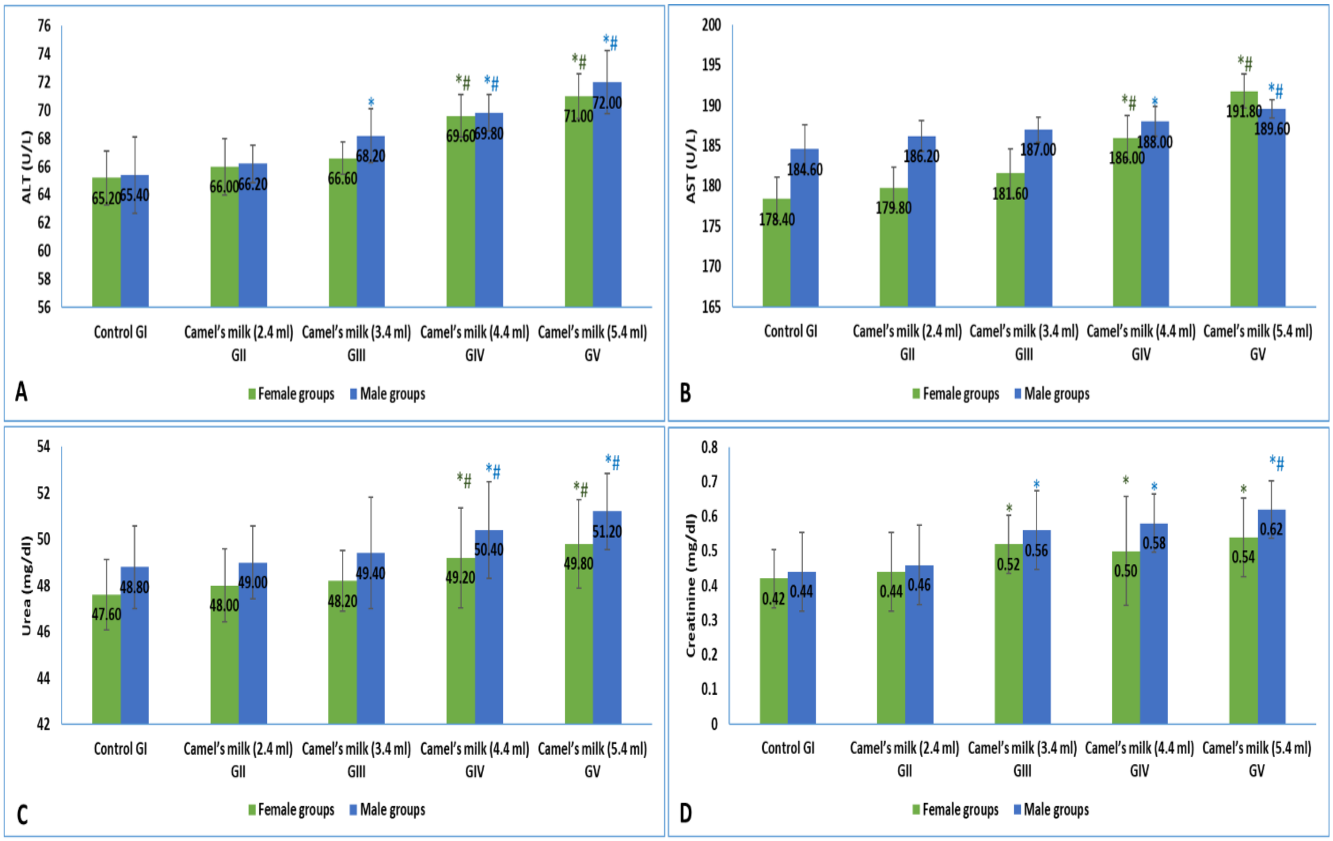

Figure S2: serum level of ALT (A), AST (B), urea (C) and creatinine (D) in different female and male animal groups. For female or male groups, results were expressed as mean±SD where * represented the significance (p<0.05) with respect to control GI and # represented the significance (p<0.05) with respect to camel's milk (3.4 mL) GIII.

4. Lipid profile:

Along all experimental groups, lipid profile (total cholesterol, triglycerides, HDL-C and LDL-C) was higher in female animals more than that of male groups.

4.1. Total cholesterol:

Female animals received 4.4 and 5.4 mL of camel's milk showed a significant high cholesterol level (70.20 and 70.80 mg/dl for GIV and GV, respectively) when compared to control GI or GIII (66.20 and 68.80 mg/dl, respectively). On the other hand, only male GV (63.80 mg/dl) showed a significant elevation in cholesterol level when compared to its corresponding GI (60.40 mg/dl) or GIII (61.60 mg/dl) (figure S3A).

4.2. Triglycerides:

Female and male GIII showed a significant elevation in triglycerides level when compared to their corresponding control GI. In female animals, only GV showed a significant elevation in triglycerides level (84.20 mg/dl) when compared to female GIII (82.20 mg/dl). On the other hand, male GIV and GV showed a significant elevation in triglycerides levels when compared to GI or GIII (figure S3B).

4.3. High density lipoprotein cholesterol (HDL-C):

In female groups, no significant difference was observed among GI, GII and GIII in HDL-C level (figure S3C). In female animals, only GV showed a significant high HDL-C level (47.80 mg/dl) when compared to female GIII (45.80 mg/dl). In male animals, GIV and GV showed no significant difference in HDL-C level when compared to male GIII.

4.4. Low density lipoprotein cholesterol (LDL-C):

No significant difference was observed in LDL-C level among all male groups (16.20, 16.60, 16.80, 17.20 and 17.60 mg/dl for GI, GII, GIII, GIV and GV, respectively). In female animals, consumption of 5.4 mL of camel's milk (GV) significantly elevated LDL-C level (18.75 mg/dl) when compared to that of GI (16.40 mg/dl) or GIII (16.75 mg/dl) (figure S3D).

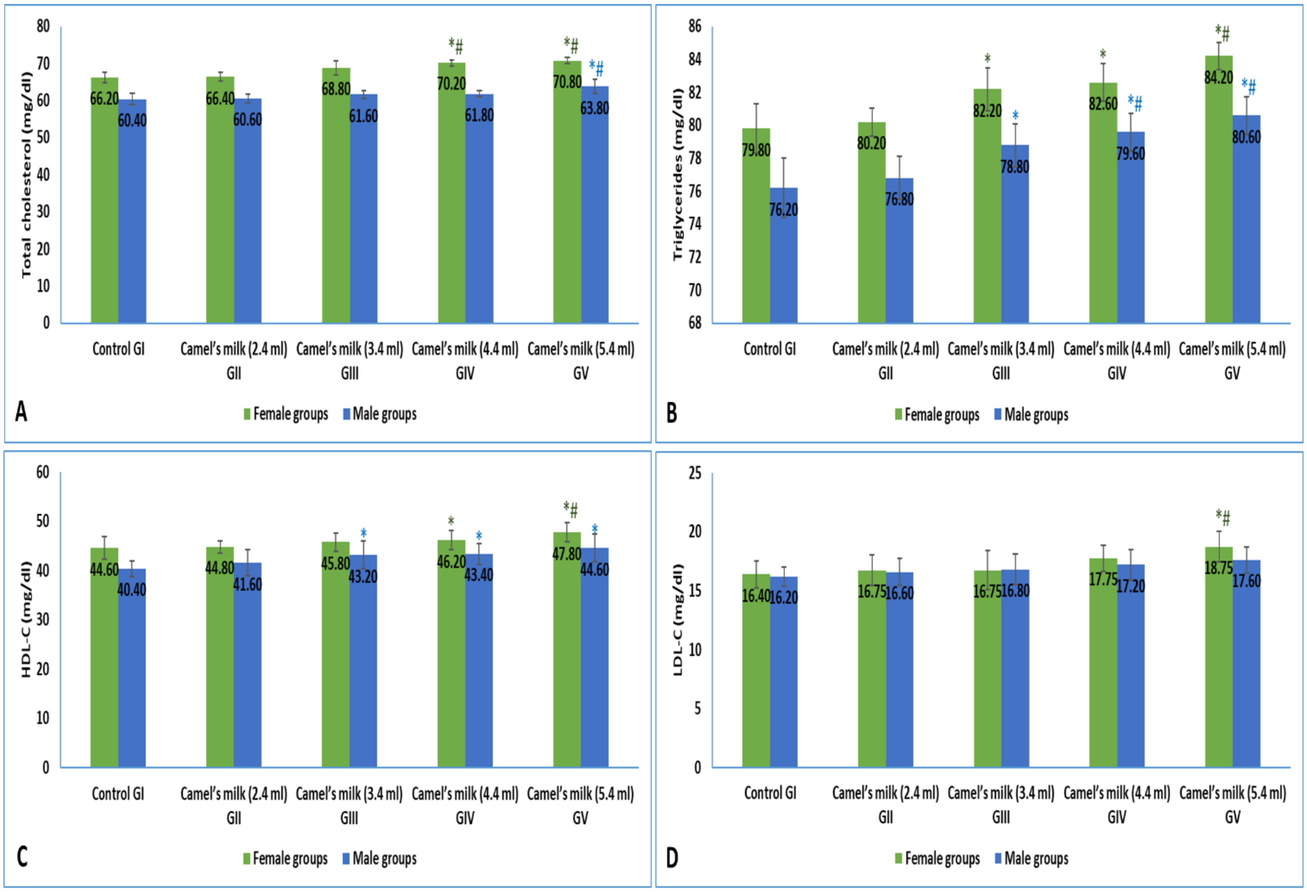

Figure S3: lipid profile in different female and male animal groups showing levels of total cholesterol (A), triglycerides (B), HDL-C (C) and LDL-C (D). For female or male groups, results were expressed as mean±SD where * represented the significance (p<0.05) with respect to control GI and # represented the significance (p<0.05) with respect to camel's milk (3.4 mL) GIII.

5. Immunological parameters:

In general, serum levels of the immunological parameters (IL-1β, IL-6, IL-17, IgM and IgG) were higher in female groups than those of male groups. IL-18 levels in female groups did not show any significant difference when compared to those of the corresponding male groups. No significant difference was observed in the rats' serum levels of the immunological parameters (IL-1β, IL-6, IL-17, IL-18, IgM and IgG) BI among different female or male groups.

5.1. IL-1β:

Serum levels of female (222.8 pg/mL) and male (202.2 pg/dl) control GI after immunization was significantly elevated than their corresponding levels BI (189.6 and 181.6 pg/mL, respectively). The same was observed in female and male GII and GIII (receiving 2.4 and 3.4 mL of camel's

milk) (table S1 and figure S4A). No significant difference was observed between the serum levels of IL-1β before and after immunization in GIV and GV in female (GIV: 195.6 Vs 191.4 and GV: 195.2 Vs 189.8 pg/mL) and male (GIV: 185.6 Vs 182.8 and GV: 183.8 Vs 182.2 pg/mL) animals. After immunization, female and male GII, GIII, GIV and GV showed a significant reduction in IL-1β level when compared to their corresponding control GI. While female GIV (195.5 pg/mL) and GV (195.2 pg/mL) showed a significant reduction in IL-1β level when compared to female GI (222.8 pg/mL), only male GV (183.8 pg/mL) showed this significant reduction with respect to male GIII (189.4 pg/mL).

5.2. IL-6:

In female groups, IL-6 level was significantly elevated after immunization (179.2 pg/mL) in GI when compared to its level before immunization (166.6 pg/mL). While female GII, GIII, GIV and GV showed no significant difference when comparing the IL-6 levels of before immunization (166.0, 165.2, 165.4 and 164.4 pg/mL, respectively) to those of after immunization (169.0, 164.4, 162.2 and 159.6 pg/mL, respectively).When comparing the female groups after immunization, GII, GIII, GIV and GV (169.0, 164.4, 162.2 and 159.6 pg/mL, respectively) showed a significant decrease in IL-6 levels when compared to control GI (179.2 pg/mL). Only female GV showed a significant reduction in IL-6 level when compared to female GIII (table S1). In all male groups, no significant difference was observed in the IL-6 level before immunization when compared to its level after immunization (figure S4B). Male GII, GIII, GIV and GV (159.8, 152.8, 152.0 and 150.8 pg/mL, respectively) showed a significant reduction in the IL-6 level when compared to that of control GI (162.4 pg/mL).

5.3. IL-17:

After immunization, female GI, GII and GIII showed a significant elevation in IL-17 level (119.4, 110.4 and 97.4 pg/mL, respectively) when compared to its level before immunization (90.8, 91.6 and 88.6 pg/mL, respectively) (figure S4C). The same behavior was observed in male GI (94.2 Vs 74.6 pg/mL), GII (89.4 Vs 76.2 pg/mL) and GIII (81.4 Vs 73.6 pg/mL). After immunization, female and male GIV and GV showed a significant reduction in IL-17 level (female: 89.4 and 89.2 pg/mL; male: 72.2 and 70.8 pg/mL) when compared to their corresponding control GI (119.4 and 94.2 pg/mL for female and male animals respectively) or GIII (97.4 and 81.4 pg/mL for female and male animals respectively). Female and male GII, GIII, GIV and GV showed a significant reduction in the IL-17 level when compared to their corresponding control GI (table S1).

5.4. IL-18:

A significant elevation in IL-18 level was observed in female and male GI (137.2 and 116.6 pg/mL, respectively) after immunization when compared to their corresponding level before immunization (103.6 and 104.8 pg/mL for female and male group, respectively). In female groups, IL-18 level in GII and GIII (120.2 and 111.6 pg/mL, respectively) after immunization were significantly higher than their corresponding levels before immunization (102.4 and 104.8 pg/mL, respectively). No significant difference was observed in IL-18 level, after immunization, of female GIV and GV when compared to their corresponding levels before immunization (table S1). In male groups, immunization with SRBCs led to a significant elevation in IL-18 level of GI only; where no change was observed in IL-18 levels upon immunization in GII, GIII, GIV or GV (figure S4D). After immunization, Female GIV and GV showed a significant reduction in IL-18 level (107.2 and 101.6 pg/mL, respectively) when compared to female GIII (111.6 pg/mL). On the other hand, male GII, GIII, GIV and GV (105.2, 103.4, 100.2 and 100.4 pg/mL, respectively) showed a significant reduction in IL-18 with respect to male GI (116.6 pg/mL).

5.5. IgM:

Immunization with SRBCs significantly elevated IgM level in female GI and GII (272.4 and 259.6 mg/L, respectively) when compared to their corresponding level before immunization (252.2 and 251.4 mg/L, respectively) (figure S4E). On the other hand, immunization in male animals led to a significant increase in IgM level in GI, GII and GIII (266.8, 252.8 and 249.6 mg/L, respectively) when compared to their corresponding level before immunization (241.0, 240.0 and 242.6 mg/L, respectively). When comparing female groups, after immunization, IgM level in GII, GIII, GIV and GV was higher than that of GI (table S1). The same was observed in male groups with the exception that GV showed a significant reduction in IgM level (240.2 mg/L) when compared to GIII (249.6 mg/L)

5.6. IgG:

In female groups, IgG level after immunization was significantly reduced in GII, GIII, GIV and GV (2.8, 2.5, 2.5 and 2.2 g/L) when compared to that of female GI (3.2 g/L). Moreover, IgG level after immunization was significantly elevated when compared to its level before immunization in female GI, GII, GIII and GIV (figure S4F). In male groups, IgG level after immunization was significantly decreased in GIII, GIV and GV (1.8, 1.7 and 1.8 g/L) when compared to that of male GI (2.1 g/L) (table S1). No significant difference was observed in IgG level after immunization in

male GIII, GIV and GV (1.8, 1.7 and 1.8 g/L, respectively) when compared to its level before immunization (1.7, 1.6 and 1.9 g/L, respectively).

Table S1: serum cytokines and immunoglobulin levels in different experimental groups BI and AI.

Item	Time	Female groups					Male groups				
		Control GI	Camel's milk (2.4 mL) GII	Camel's milk (3.4 mL) GIII	Camel's milk (4.4 mL) GIV	Camel's milk (5.4 mL) GV	Control GI	Camel's milk (2.4 mL) GII	Camel's milk (3.4 mL) GIII	Camel's milk (4.4 mL) GIV	Camel's milk (5.4 mL) GV
**IL-1β (pg/mL)**	**BI**	189.6±1.1	189.0±4.1	189.4±4.9	191.4±3.9	189.8±3.5	181.6±3.6	181.6±5.1	180.4±2.6	182.8±3.2	182.2±2.2
	**AI**	222.8±2.8	213.6±3.9*	202.8±3.5*	195.6±2.7*#	195.2±1.9*#	202.2±1.9	194.6±6.8*	189.4±6.0*	185.6±3.2*	183.8±3.3*#
**IL-6 (pg/mL)**	**BI**	166.6±3.3	166.0±3.6	165.2±3.5	165.4±2.9	164.4±3.0	159.8±6.9	158.2±3.1	153.0±4.1	152.0±4.4	152.6±3.5
	**AI**	179.2±3.1	169.0±7.3*	164.4±1.9*	162.2±3.7*	159.6±4.5*#	162.4±3.0	159.8±1.9*	152.8±4.7*	152.0±3.3*	150.8±1.3*
**IL-17 (pg/mL)**	**BI**	90.8±1.6	91.6±2.3	88.6±4.4	88.0±2.5	89.4±7.5	74.6±3.8	76.2±4.2	73.6±3.3	75.4±3.1	72.8±3.5
	**AI**	119.4±2.4	110.4±4.0*	97.4±3.0*	89.4±4.4*#	89.2±2.7*#	94.2±5.4	89.4±2.7*	81.4±2.9*	72.2±2.7*#	70.8±1.6*#
**IL-18 (pg/mL)**	**BI**	103.6±2.8	102.4±5.2	104.8±5.9	102.6±7.2	103.2±5.8	104.8±3.7	103.2±7.3	102.6±7.1	100.8±8.1	101.0±4.9
	**AI**	137.2±4.3	120.2±3.8*	111.6±6.1*	107.2±2.7*#	101.6±5.1*#	116.6±3.6	105.2±6.6*	103.4±4.7*	100.2±7.9*	100.4±10.8*
**IgM (mg/L)**	**BI**	252.2±4.3	251.4±3.7	250.4±1.5	249.8±3.1	247.4±3.2	241.0±2.9	240.4±2.8	242.6±5.8	241.8±2.9	240.8±1.6
	**AI**	272.4±3.3	259.6±1.8*	251.8±4.9*	251.4±2.8*	250.2±1.6*	266.8±5.1	252.8±4.9*	249.6±4.3*	242.0±3.4*	240.2±3.9*#
**IgG (g/L)**	**BI**	2.1±0.3	2.1±0.3	2.2±0.5	2.1±0.3	2.2±0.4	1.4±0.3	1.3±0.4	1.7±0.4	1.6±0.5	1.9±0.6
	**AI**	3.2±0.3	2.8±0.2*	2.5±0.3*	2.5±0.3*	2.2±0.4*	2.1±0.3	1.9±0.2	1.8±0.5*	1.7±0.4*	1.8±0.3*

For female or male groups, results were expressed as mean±SD where * represented the significance ($p<0.05$) with respect to control GI and # represented the significance ($p<0.05$) with respect to camel's milk (3.4 mL) GIII.

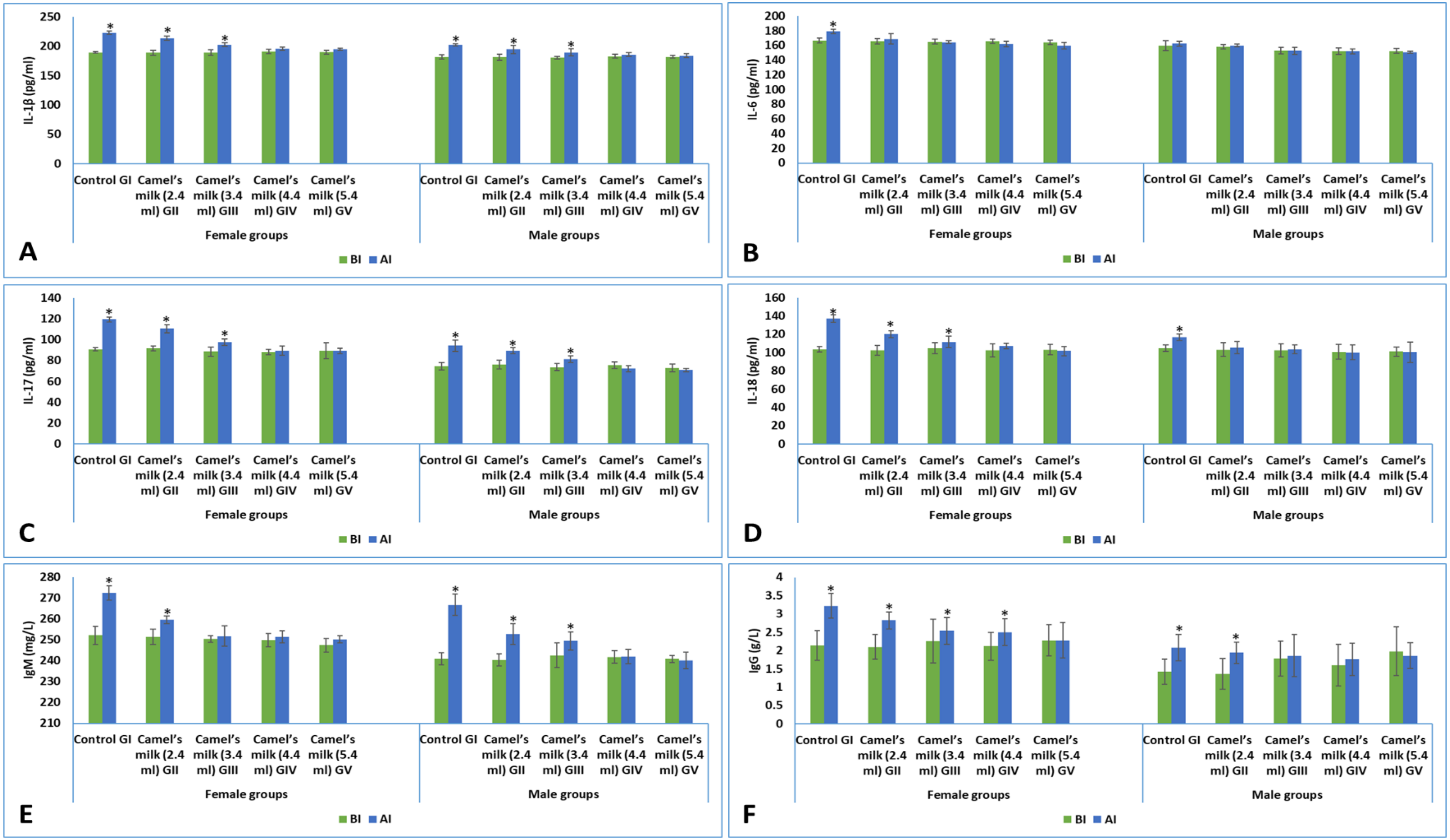

Figure S4: immunological parameters in different female and male animal groups BI and AI showing levels of IL-1β, IL-6, IL-17, IL-18, IgM and IgG. For each female or male groups, results were expressed as mean±SD where * represented the significance ($p<0.05$) with respect to BI level.

6. Dose-response effects of camel milk on body weight of weaning rats:

The analysis of body weight revealed a complex, dose-dependent effect of camel milk supplementation that was modulated by sex (table S2). In female rats, low-to-moderate doses of camel milk were associated with enhanced growth. The GII (2.4 mL) and GIII (3.4 mL) groups showed a significant increase in final body weight and weight gain compared to the control group. However, this beneficial trend was reversed at higher doses. Females in the GIV (4.4 mL) and GV (5.4 mL) groups exhibited a significant decrease in final weight and weight gain not only compared to the control but also relative to the optimal GIII dose. A similar pattern was observed in male rats, though the response was less pronounced. The GIII group showed a significant improvement in final weight and weight gain compared to the control. Conversely, the GV group demonstrated a significant reduction in final weight compared to the control, and both the GIV and GV groups showed significantly lower final weight and weight gain relative to the GIII group ($p < 0.05$). These results indicated that while the WHO-equivalent dose (GIII, 3.4 mL) promoted healthy growth, supra-physiological doses (4.4 mL and 5.4 mL) had a suppressive effect on weight gain in both sexes, with females being more susceptible.

Table S2: Body weight parameters of weaning rats administered different doses of camel milk for six weeks.

Group	Sex	Initial Weight (g)	Final Weight (g)	Weight Gain (g)
**Control (GI)**	**Female**	71.6±2.7	215.0±2.2	143.4±4.7
	**Male**	72.8±3.7	301.6±2.7	228.8±5.1
**GII (2.4 mL)**	**Female**	72.4±4.9	221.2±1.9*	148.8±4.4
	**Male**	73.6±3.9	307.2±2.5	233.6±6.1
**GIII (3.4 mL)**	**Female**	73.4±3.8	226.4±3.5$^{*\#}$	153.0±5.7
	**Male**	74.6±6.1	313.8±3.2*	239.2±6.8*
**GIV (4.4 mL)**	**Female**	72.0±4.5	208.4±2.4$^{*\#}$	136.4±4.3$^{\#}$
	**Male**	72.4±2.7	298.6±3.8$^{\#}$	226.2±4.3$^{\#}$
**GV (5.4 mL)**	**Female**	72.2±4.8	202.6±2.7$^{*\#}$	130.4±6.9$^{*\#}$
	**Male**	72.2±2.5	292.0±3.1$^{*\#}$	219.8±3.2$^{\#}$

For female or male groups, in each column, results were expressed as mean±SD where * represented the significance (p<0.05) with respect to control GI and # represented the significance (p<0.05) with respect to camel's milk (3.4 mL) GIII.

The administration of camel milk exerted a distinct biphasic, dose-dependent effect on the growth performance of female weaning rats (table S3). All treatment groups began the study with initial body weights that were not significantly different from the control group, confirming a valid baseline. However, divergent outcomes emerged by the end of the six-week intervention. Rats receiving the lower doses of camel milk demonstrated enhanced growth. The GII group (2.4 mL) showed a significant and large increase in final body weight compared to controls (Mean Diff. = +6.2, 95% CI [3.16, 9.24], Cohen's d = 2.97). This beneficial effect was most pronounced in the GIII group (3.4 mL, WHO-equivalent), which exhibited a very large, significant increase in both final body weight (Mean Diff. = +11.4, 95% CI [6.94, 15.86], Cohen's d = 3.82) and total weight gain (Mean Diff. = +9.6, 95% CI [1.97, 17.23], Cohen's d = 1.83), establishing this dose as optimal for promoting growth. In contrast, supra-physiological doses were detrimental. The GIV group (4.4 mL) displayed a significant decrease in final body weight and weight gain (Mean Diff. = -7.0, 95% CI [-13.61, -0.39], Cohen's d = -1.54), indicating the onset of growth suppression. This adverse effect was amplified in the GV group (5.4 mL), which suffered a substantial and very large impairment in growth, with significant reductions in both final body weight (Mean Diff. = -12.4, 95% CI [-16.02, -8.78], Cohen's d = -5.00) and weight gain (Mean Diff. = -13.0, 95% CI [-21.89, -4.11], Cohen's d = -2.18). Collectively, these results delineated a clear therapeutic window for camel milk, where low-to-moderate doses were growth-promoting, but high doses were growth-inhibiting in female weaning rats.

A similar biphasic, dose-dependent response to camel milk supplementation was observed in male weaning rats, though with a slightly higher tolerance for the highest dose compared to females (table S4). All groups were well-matched at baseline, with no significant differences in initial body weight. The lower and recommended doses of camel milk demonstrated significant growth-promoting effects. The GII group (2.4 mL) exhibited a significant and large increase in final body weight (Mean Diff. = +5.6, 95% CI [1.74, 9.46], Cohen's d = 2.11). The most substantial benefit was again observed in the GIII group (3.4 mL, WHO-equivalent), which showed a very large, significant increase in both final body weight (Mean Diff. = +12.2, 95% CI [7.82, 16.58], Cohen's d = 4.06) and total weight gain (Mean Diff. = +10.4, 95% CI [1.53, 19.27], Cohen's d = 1.71), confirming this as the optimal dose for enhancing growth in males. The adverse effects of higher doses in males manifested at a clearer threshold. Unlike in females, the GIV group (4.4 mL) showed no statistically significant effect on final weight or weight gain, although the negative mean differences indicated a trending suppression. However, the GV group (5.4 mL) displayed a significant and large detrimental impact, with

substantial reductions in both final body weight (Mean Diff. = -9.6, 95% CI [-13.89, -5.31], Cohen's d = -3.26) and weight gain (Mean Diff. = -9.0, 95% CI [-15.47, -2.53], Cohen's d = -2.08). These findings confirmed that the WHO-equivalent dose (3.4 mL) was optimal for male rats, while the very high dose (5.4 mL) consistently impaired growth, with males potentially exhibiting marginally greater resilience to the suppressive effects of the 4.4 mL dose compared to females.

Table S3: Dose-response effects of camel milk on biochemical, hepatic, renal, and metabolic parameters in female weaning rats.

Parameter	Group	Mean diff.	95% CI	Cohen's d	Significance & interpretation
**Initial Weight (g)**	GII	+0.8	[-5.35, +6.95]	0.20	Not significant
	GIII	+1.80	[-3.17, +6.77]	0.54	Not significant
	GIV	+0.40	[-5.18, +5.98]	0.11	Not significant
	GV	+0.6	[-5.44, +6.64]	0.15	Not significant
**Final Weight (g)**	GII	+6.2	[+3.16, +9.24]	2.97	Significant increase. Small dose promoted healthy growth.
	GIII	+11.4	[+6.94, +15.86]	3.82	Significant increase. WHO-equivalent dose optimally enhanced growth
	GIV	-6.6	[-9.99, -3.21]	-2.84	Significant decrease. High dose began to suppress growth
	GV	-12.4	[-16.02, -8.78]	-5	Significant decrease. Very high dose strongly impaired growth.
**Weight Gain (g)**	GII	+5.4	[-1.28, +12.08]	1.17	Not significant
	GIII	+9.6	[+1.97, +17.23]	1.83	Significant increase. Confirms a clear growth-promoting effect.
	GIV	-7	[-13.61, -0.39]	-1.54	Significant decrease. High dose significantly reduced weight gain.
	GV	-13	[-21.89, -4.11]	-2.18	Significant decrease. Very high dose caused substantial growth impairment.

Data were presented as the mean difference from the control group (GI), 95% confidence interval (95% CI), and effect size (Cohen's d). Statistical significance and interpretation were determined as follows: a result was considered statistically significant if its 95% CI did not include zero. The direction of the effect (beneficial or adverse) was assigned based on the known physiology of the parameter. The magnitude of the effect size was interpreted using Cohen's criteria: $|d| \geq 0.8$ represents a large effect, and $|d| \geq 2.0$ represents a very large effect.

Table S4: Dose-response effects of camel milk on biochemical, hepatic, renal, and metabolic parameters in male weaning rats.

Parameter	Group	Mean diff.	95% CI	Cohen's d	Significance & interpretation
**Initial Weight (g)**	GII	+0.8	[-4.75, +6.35]	0.21	Not significant
	GIII	+1.8	[-5.75, +9.35]	0.36	Not significant
	GIV	-0.4	[-5.25, +4.45]	-0.12	Not significant
	GV	-0.6	[-5.38, +4.18]	-0.19	Not significant
**Final Weight (g)**	GII	+5.6	[+1.74, +9.46]	2.11	Significant increase. Small dose promoted healthy growth.
	GIII	+12.2	[+7.82, +16.58]	4.06	Significant increase. WHO-equivalent dose optimally enhanced growth.
	GIV	-3	[-7.97, +1.97]	-0.9	Not significant
	GV	-9.6	[-13.89, -5.31]	-3.26	Significant decrease. Very high dose strongly impaired growth.
**Weight Gain (g)**	GII	+4.8	[-3.42, +13.02]	0.85	Not significant
	GIII	+10.4	[+1.53, +19.27]	1.71	Significant increase. Confirms a clear growth-promoting effect on this dose.
	GIV	-2.6	[-9.55, +4.35]	-0.54	Not significant
	GV	-9	[-15.47, -2.53]	-2.08	Significant decrease. Very high dose caused substantial growth impairment.

Data were presented as the mean difference from the control group (GI), 95% confidence interval (95% CI), and effect size (Cohen's d). Statistical significance and interpretation were determined as follows: a result was considered statistically significant if its 95% CI did not include zero. The direction of the effect (beneficial or adverse) was assigned based on the known physiology of the parameter. The magnitude of the effect size was interpreted using Cohen's criteria: $|d| \geq 0.8$ represents a large effect, and $|d| \geq 2.0$ represents a very large effect.

7. **Dose-response effects of camel milk on biochemical, hepatic, renal, and metabolic parameters:**

In female weaning rats, camel milk administration exerted pronounced, dose-dependent effects on a wide range of biochemical parameters (table S5). Bone metabolism was significantly enhanced, as evidenced by large, dose-dependent increases in total calcium (e.g., GV: Cohen's d = 6.07) and its active hormonal form, calcitriol (e.g., GIV: Cohen's d = 4.57). This was supported by a substantial rise in the vitamin D precursor, calcifediol, across all treatment groups. A clear threshold for hepatic impact was observed; liver enzymes (ALT, AST) were significantly elevated only at the 4.4 mL and 5.4 mL doses, indicating hepatic stress at these higher intakes (e.g., GV AST: Cohen's d = 5.47). The lipid profile was similarly affected in a dose-dependent manner, with significant increases in total cholesterol, triglycerides, and LDL-C at higher doses, demonstrating a general hyperlipidemic effect. Notably, the 3.4 mL dose (the WHO-equivalent) consistently showed significant beneficial effects on bone and vitamin D metrics without inducing the significant hepatic and lipid stress associated with the two highest doses. Renal parameters (urea, creatinine) remained largely unaffected across all doses.

In male weaning rats, camel milk administration produced a distinct dose-response profile characterized by strong benefits to bone metabolism and vitamin D status, alongside emerging metabolic stress at higher doses (table S6). Bone health was markedly enhanced, with the 3.4 mL dose and above inducing very large, significant increases in total calcium (e.g., GIV: Cohen's d = 7.92) and its regulatory hormones, calcifediol and calcitriol. A notable sex difference was observed in the hepatic response; while significant liver stress (elevated ALT and AST) was detected at the 4.4 mL and 5.4 mL doses, the effect sizes in males were generally smaller than those observed in females. The lipid profile showed a clear adverse trend, with triglycerides significantly elevated from the 3.4 mL dose, and total cholesterol significantly increased at the 5.4 mL dose. In contrast to the lipid fractions, HDL-C was significantly improved at the two highest doses, indicating a mixed lipid response. A key finding in males was a significant dose-dependent increase in serum creatinine, a marker of renal function, which became significant at the 5.4 mL dose, suggesting a renal burden not seen in females. Overall, the 3.4 mL dose provided substantial benefits for bone and vitamin D metabolism with minimal adverse effects, while the two highest doses presented a trade-off between these benefits and significant hepatic, renal, and lipid stress.

Table S5: Dose-response effects of camel milk on biochemical, hepatic, renal, and metabolic parameters in female weaning rats.

Parameter	Group	Mean diff.	95% CI	Cohen's d	Significance & interpretation
**Ionized Ca**	GII	+0.03	[-0.18, +0.25]	0.23	No effect
	GIII	+0.23	[-0.01, +0.47]	1.42	Marginal benefit
	GIV	+0.27	[+0.05, +0.48]	1.88	Beneficial effect
	GV	+0.35	[+0.10, +0.61]	2.04	Beneficial effect
**Total Ca**	GII	+0.24	[+0.01, +0.47]	1.55	Beneficial effect
	GIII	+0.82	[+0.61, +1.03]	5.80	Large benefit
	GIV	+1.08	[+0.86, +1.30]	7.12	Very large benefit
	GV	+1.82	[+1.33, +2.31]	6.07	Very large benefit
**Phosphorus**	GII	-0.08	[-0.53, +0.37]	-0.27	No effect
	GIII	+0.04	[-0.32, +0.40]	0.16	No effect
	GIV	+0.38	[+0.10, +0.66]	2.00	Beneficial effect
	GV	+0.24	[-0.09, +0.57]	1.07	No effect
**Calcifediol**	GII	+3.40	[+0.50, +6.30]	1.82	Beneficial effect
	GIII	+7.40	[+4.40, +10.40]	3.82	Large benefit
	GIV	+11.00	[+8.30, +13.70]	6.30	Very large benefit
	GV	+12.80	[+9.07, +16.53]	5.59	Very large benefit
**Calcitriol**	GII	-0.40	[-4.64, +3.84]	-0.14	No effect
	GIII	+3.20	[-0.37, +6.77]	1.31	No effect
	GIV	+9.80	[+6.59, +13.01]	4.57	Very large benefit
	GV	+13.40	[+8.33, +18.47]	3.95	Large benefit
**ALT**	GII	+0.80	[-2.06, +3.66]	0.41	No effect
	GIII	+1.40	[-0.96, +3.76]	0.89	No effect
	GIV	+4.40	[+1.87, +6.93]	2.54	Adverse effect (hepatic stress)
	GV	+5.80	[+3.23, +8.37]	3.29	Adverse effect (hepatic stress)
**AST**	GII	+1.40	[-2.46, +5.26]	0.53	No effect
	GIII	+3.20	[-1.00, +7.40]	1.11	No effect
	GIV	+7.60	[+3.63, +11.57]	2.79	Adverse effect (hepatic stress)
	GV	+13.40	[+9.83, +16.97]	5.47	Strong adverse effect (hepatic stress)

**Urea**	GII	+0.40	[-1.86, +2.66]	0.26	No effect
	GIII	+0.60	[-1.46, +2.66]	0.42	No effect
	GIV	+1.60	[-1.20, +4.40]	0.86	No effect
	GV	+2.20	[-0.33, +4.73]	1.27	No effect
**Creatinine**	GII	+0.02	[-0.13, +0.17]	0.20	No effect
	GIII	+0.10	[-0.02, +0.22]	1.20	No effect
	GIV	+0.08	[-0.12, +0.28]	0.63	No effect
	GV	+0.12	[-0.03, +0.27]	1.20	No effect
**Total Cholesterol**	GII	+0.20	[-1.59, +1.99]	0.16	No effect
	GIII	+2.60	[+0.14, +5.06]	1.58	Adverse effect
	GIV	+4.00	[+2.36, +5.64]	3.65	Adverse effect
	GV	+4.60	[+2.96, +6.24]	4.20	Adverse effect
**Triglycerides**	GII	+0.40	[-1.46, +2.26]	0.33	No effect
	GIII	+2.40	[+0.36, +4.44]	1.72	Adverse effect
	GIV	+2.80	[+0.87, +4.73]	2.12	Adverse effect
	GV	+4.40	[+2.54, +6.26]	3.65	Adverse effect
**HDL-C**	GII	+0.20	[-2.70, +3.10]	0.11	No effect
	GIII	+1.20	[-1.89, +4.29]	0.57	No effect
	GIV	+1.60	[-1.49, +4.69]	0.75	No effect
	GV	+3.20	[+0.11, +6.29]	1.51	Beneficial effect
**LDL-C**	GII	+0.40	[-1.39, +2.19]	0.29	No effect
	GIII	+1.00	[-1.14, +3.14]	0.24	No effect
	GIV	+1.20	[-0.46, +2.86]	1.18	No effect
	GV	+2.80	[+1.01, +4.59]	1.92	Adverse effect

Data were presented as the mean difference from the control group (GI), 95% confidence interval (95% CI), and effect size (Cohen's d). Statistical significance and interpretation were determined as follows: a result was considered statistically significant if its 95% CI did not include zero. The direction of the effect (beneficial or adverse) was assigned based on the known physiology of the parameter. The magnitude of the effect size was interpreted using Cohen's criteria: $|d| \geq 0.8$ represents a large effect, and $|d| \geq 2.0$ represents a very large effect.

Table S6: Dose-response effects of camel milk on biochemical, hepatic, renal, and metabolic parameters in male weaning rats.

Parameter	Group	Mean diff.	95% CI	Cohen's d	Significance & interpretation
**Ionized Ca**	GII	-0.02	[-0.17, +0.13]	-0.20	No effect
	GIII	+0.12	[-0.08, +0.32]	0.95	No effect
	GIV	+0.26	[+0.11, +0.41]	2.60	Beneficial effect
	GV	+0.24	[-0.04, +0.52]	1.40	No effect
**Total Ca**	GII	+0.10	[-0.31, +0.51]	0.36	No effect
	GIII	+0.96	[+0.58, +1.34]	4.13	Large benefit
	GIV	+1.68	[+1.31, +2.05]	7.92	Very large benefit
	GV	+1.86	[+1.44, +2.28]	6.62	Very large benefit
**Phosphorus**	GII	+0.08	[-0.47, +0.63]	0.22	No effect
	GIII	+0.28	[-0.13, +0.69]	1.00	No effect
	GIV	+0.40	[+0.05, +0.75]	1.66	Beneficial effect
	GV	+0.36	[+0.05, +0.67]	1.80	Beneficial effect
**Calcifediol**	GII	+0.20	[-2.89, +3.29]	0.09	No effect
	GIII	+5.60	[+2.70, +8.50]	2.89	Large benefit
	GIV	+9.60	[+6.70, +12.50]	5.13	Very large benefit
	GV	+9.60	[+5.11, +14.09]	3.20	Large benefit
**Calcitriol**	GII	+0.20	[-3.70, +4.10]	0.08	No effect
	GIII	+7.40	[+4.92, +9.88]	4.35	Large benefit
	GIV	+9.00	[+6.47, +11.53]	5.20	Very large benefit
	GV	+12.00	[+9.10, +14.90]	6.20	Very large benefit
**ALT**	GII	+0.80	[-2.48, +4.08]	0.38	No effect
	GIII	+2.80	[-0.71, +6.31]	1.19	No effect
	GIV	+4.40	[+1.12, +7.68]	2.07	Adverse effect (hepatic stress)
	GV	+6.60	[+2.98, +10.22]	2.66	Adverse effect (hepatic stress)
**AST**	GII	+1.60	[-2.14, +5.34]	0.64	No effect
	GIII	+2.40	[-1.28, +6.08]	1.01	No effect
	GIV	+3.40	[-0.31, +7.11]	1.37	No effect
	GV	+5.00	[+1.35, +8.65]	2.22	Adverse effect (hepatic stress)

**Urea**	GII	+0.20	[-2.26, +2.66]	0.12	No effect
	GIII	+0.60	[-2.57, +3.77]	0.19	No effect
	GIV	+1.60	[-1.22, +4.42]	0.52	No effect
	GV	+2.40	[-0.10, +4.90]	0.47	No effect
**Creatinine**	GII	+0.02	[-0.15, +0.19]	0.18	No effect
	GIII	+0.12	[-0.05, +0.29]	1.05	No effect
	GIV	+0.14	[-0.01, +0.29]	1.40	Marginal adverse effect
	GV	+0.18	[+0.03, +0.33]	1.80	Adverse effect
**Total Cholesterol**	GII	+0.20	[-1.81, +2.21]	0.15	No effect
	GIII	+1.20	[-0.81, +3.21]	0.89	No effect
	GIV	+1.40	[-0.50, +3.30]	1.14	No effect
	GV	+3.40	[+0.87, +5.93]	1.96	Adverse effect
**Triglycerides**	GII	+0.60	[-1.74, +2.94]	0.38	No effect
	GIII	+2.60	[+0.26, +4.94]	1.66	Adverse effect
	GIV	+3.40	[+1.16, +5.64]	2.27	Adverse effect
	GV	+4.40	[+2.16, +6.64]	2.93	Adverse effect
**HDL-C**	GII	+1.20	[-2.19, +4.59]	0.55	No effect
	GIII	+2.80	[-0.66, +6.26]	1.25	No effect
	GIV	+3.00	[+0.28, +5.72]	1.65	Beneficial effect
	GV	+4.20	[+0.64, +7.76]	1.82	Beneficial effect
**LDL-C**	GII	+0.40	[-1.10, +1.90]	0.40	No effect
	GIII	+0.60	[-1.04, +2.24]	0.55	No effect
	GIV	+1.00	[-0.64, +2.64]	0.91	No effect
	GV	+1.40	[-0.10, +2.90]	1.40	No effect

Data were presented as the mean difference from the control group (GI), 95% confidence interval (95% CI), and effect size (Cohen's d). Statistical significance and interpretation were determined as follows: a result was considered statistically significant if its 95% CI did not include zero. The direction of the effect (beneficial or adverse) was assigned based on the known physiology of the parameter. The magnitude of the effect size was interpreted using Cohen's criteria: $|d| \geq 0.8$ represents a large effect, and $|d| \geq 2.0$ represents a very large effect.

8. Dose-dependent effects of camel milk on cytokines and immunoglobulins levels after immunization with SRBCs:

Camel milk administration demonstrated potent, dose-dependent immunomodulatory effects on the pro-inflammatory cytokine response in female weaning rats following SRBC immunization (table S7). The composite pro-inflammatory cytokine score revealed significant suppression across all treatment groups, with effect sizes increasing progressively from moderate at the 2.4 mL dose to very large at the 5.4 mL dose. Analysis of individual cytokines showed that IL-1β levels were significantly reduced at all doses (95% CIs: -14.33 to -4.07 for GII; -24.71 to -15.29 for GIII; -31.26 to -23.14 for GIV; -31.25 to -23.95 for GV), with particularly strong suppression observed at higher doses. Similarly, IL-6 demonstrated significant dose-dependent suppression beginning at the 3.4 mL dose (95% CI: -18.76 to -10.84), while IL-17 showed substantial suppression across all doses with the strongest effect at the 5.4 mL dose (95% CI: -33.99 to -26.41). IL-18 exhibited a distinct response pattern, with significant suppression only at higher doses (4.4 mL and 5.4 mL). These findings collectively demonstrated that camel milk exerted a strong, dose-responsive anti-inflammatory effect in female rats by significantly attenuating the pro-inflammatory cytokine cascade following immune challenge. Camel milk administration significantly modulated the humoral immune response in a dose- and sex-dependent manner following SRBC immunization. In female rats, the composite humoral immunity score revealed a significant and progressive suppressive effect, with effect sizes ranging from moderate at the 3.4 mL dose to large at the 5.4 mL dose. Analysis of individual immunoglobulins showed that both IgM and IgG levels were significantly reduced in females receiving the 4.4 mL and 5.4 mL doses (e.g., Female GV IgM: 95% CI [-26.29, -18.11]; Female GV IgG: 95% CI [-1.56, -0.32]).

Camel milk administration in male weaning rats demonstrated a clear dose-dependent immunomodulatory effect following SRBC immunization, though with a different profile than observed in females (table S8). The pro-inflammatory cytokine response was significantly attenuated, with IL-1β suppression becoming significant at the 3.4 mL dose and IL-6 suppression starting at the same dose, both showing very large effect sizes (e.g., GIV IL-1β Cohen's d = -6.27). IL-17 was also significantly suppressed from the 3.4 mL dose onwards, while IL-18 was significantly reduced at all administered doses. The most pronounced effect was observed on the humoral immune response; IgM levels were significantly and potently suppressed across all treatment groups with very large effect sizes (e.g., GV IgM Cohen's d = -5.85). In contrast, IgG levels in male rats remained completely unaffected by camel milk administration at any dose, showing no significant differences from the control group. This pattern indicated that camel milk exerted a potent, dose-dependent anti-

inflammatory effect on the cytokine cascade and a specific, strong suppressive effect on the early antibody response (IgM) in male rats, while leaving the later, adaptive antibody response (IgG) intact.

Table S7: Dose-dependent effects of camel milk on cytokines and immunoglobulins levels in female weaning rats after SRBC immunization.

Parameter	Group	Mean diff.	95% CI	Cohen's d	Significance & interpretation
**IL-1β**	GII	-9.20	[-14.33, -4.07]	-2.68	Strong suppression
	GIII	-20.00	[-24.71, -15.29]	-6.19	Potent suppression
	GIV	-27.20	[-31.26, -23.14]	-9.77	Potent suppression
	GV	-27.60	[-31.25, -23.95]	-11.31	Potent suppression
**IL-6**	GII	-10.20	[-19.41, -0.99]	-1.80	Significant suppression
	GIII	-14.80	[-18.76, -10.84]	-5.59	Potent suppression
	GIV	-17.00	[-22.09, -11.91]	-4.87	Potent suppression
	GV	-19.60	[-25.44, -13.76]	-5.02	Potent suppression
**IL-17**	GII	-9.00	[-13.97, -4.03]	-2.71	Strong suppression
	GIII	-22.00	[-26.01, -17.99]	-8.01	Potent suppression
	GIV	-30.00	[-35.54, -24.46]	-8.39	Potent suppression
	GV	-30.20	[-33.99, -26.41]	-11.62	Potent suppression
**IL-18**	GII	-17.00	[-22.96, -11.04]	-4.16	Significant suppression
	GIII	-25.60	[-33.51, -17.69]	-4.84	Potent suppression
	GIV	-30.00	[-35.43, -24.57]	-8.26	Potent suppression
	GV	-35.60	[-42.56, -28.64]	-7.46	Potent suppression
**IgM**	GII	-12.80	[-16.98, -8.62]	-4.74	Strong suppression
	GIII	-20.60	[-26.90, -14.30]	-4.89	Potent suppression
	GIV	-21.00	[-25.57, -16.43]	-6.71	Potent suppression
	GV	-22.20	[-26.29, -18.11]	-8.39	Potent suppression
**IgG**	GII	-0.40	[-0.83, +0.03]	-1.38	No significant Effect
	GIII	-0.68	[-1.19, -0.17]	-1.94	Significant suppression
	GIV	-0.72	[-1.23, -0.21]	-2.05	Significant suppression
	GV	-0.94	[-1.56, -0.32]	-2.27	Significant suppression

Data were presented as mean difference from control group (GI), 95% confidence interval (95% CI), and effect size (Cohen's d). Statistical significance was determined when 95% CI did not include zero. Negative values indicate suppression of cytokine/immunoglobulin levels compared to control. The magnitude of the effect size was interpreted using Cohen's criteria: $|d| \geq 0.8$ represents a large effect, and $|d| \geq 2.0$ represents a very large effect.

Table S8: Dose-dependent effects of camel milk on cytokines and immunoglobulins levels in male weaning rats after SRBC immunization.

Parameter	Group	Mean diff.	95% CI	Cohen's d	Significance & interpretation
**IL-1β**	GII	-7.60	[-15.73, +0.53]	-1.52	No significant Effect
	GIII	-12.80	[-20.07, -5.53]	-2.86	Strong suppression
	GIV	-16.60	[-20.56, -12.64]	-6.27	Potent suppression
	GV	-18.40	[-22.62, -14.18]	-6.74	Potent suppression
**IL-6**	GII	-2.60	[-6.41, +1.21]	-1.02	No significant Effect
	GIII	-9.60	[-15.54, -3.66]	-2.42	Significant suppression
	GIV	-10.40	[-15.10, -5.70]	-3.22	Strong suppression
	GV	-11.60	[-15.41, -7.79]	-4.95	Potent suppression
**IL-17**	GII	-4.80	[-11.41, +1.81]	-1.12	No significant Effect
	GIII	-12.80	[-19.55, -6.05]	-1.84	Significant suppression
	GIV	-22.00	[-28.65, -15.35]	-2.14	Strong suppression
	GV	-23.40	[-29.89, -16.91]	-0.35	Strong suppression
**IL-18**	GII	-11.40	[-19.70, -3.10]	-2.13	Significant suppression
	GIII	-13.20	[-19.35, -7.05]	-3.13	Strong suppression
	GIV	-16.40	[-25.94, -6.86]	-2.66	Strong suppression
	GV	-16.20	[-29.39, -3.01]	-2.00	Significant suppression
**IgM**	GII	-14.00	[-21.32, -6.68]	-2.79	Strong suppression
	GIII	-17.20	[-24.08, -10.32]	-3.65	Strong suppression
	GIV	-24.80	[-31.29, -18.31]	-5.71	Potent suppression
	GV	-26.60	[-33.24, -19.96]	-5.85	Potent suppression
**IgG**	GII	-0.14	[-0.62, +0.34]	-0.43	No significant Effect
	GIII	-0.22	[-0.94, +0.50]	-0.46	No significant Effect
	GIV	-0.32	[-0.91, +0.27]	-0.79	No significant Effect
	GV	-0.22	[-0.74, +0.30]	-0.62	No significant Effect

Data were presented as mean difference from control group (GI), 95% confidence interval (95% CI), and effect size (Cohen's d). Statistical significance was determined when 95% CI did not include zero. Negative values indicate suppression of cytokine/immunoglobulin levels compared to control. The magnitude of the effect size was interpreted using Cohen's criteria: $|d| \geq 0.8$ represents a large effect, and $|d| \geq 2.0$ represents a very large effect.

9. Histopathological examination:

Histological examination of key organs provided crucial morphological correlation with the biochemical and immunological findings. Spleen sections from the control (GI), low-dose (GII), and optimal-dose (GIII) groups were largely comparable, showing average architecture with small lymphoid follicles and an expanded red pulp (figure S5). In contrast, the high-dose groups (GIV and GV) exhibited improved lymphoid follicle definition but also marked congestion of the blood sinusoids and scattered apoptotic lymphocytes, indicating immune activation and stress. Liver histology revealed a clear dose-dependent effect (figure S6). Tissues from the GI, GII, and GIII groups appeared normal with minimal inflammation. However, the high-dose groups (GIV and GV) displayed significant pathological changes, including moderate portal inflammatory infiltrate, marked dilation and congestion of the portal vein, and extensive hepatocyte apoptosis, confirming the biochemical evidence of hepatic stress. Kidney sections from all groups, including the high-dose cohorts, showed preserved architecture with average glomeruli and tubules (figure S7). Only scattered, minimal apoptotic cells were observed in the tubular epithelium, indicating an absence of overt structural damage. This aligns with the biochemical data, suggesting that the elevated creatinine in high-dose males reflected functional stress rather than irreversible histological injury.

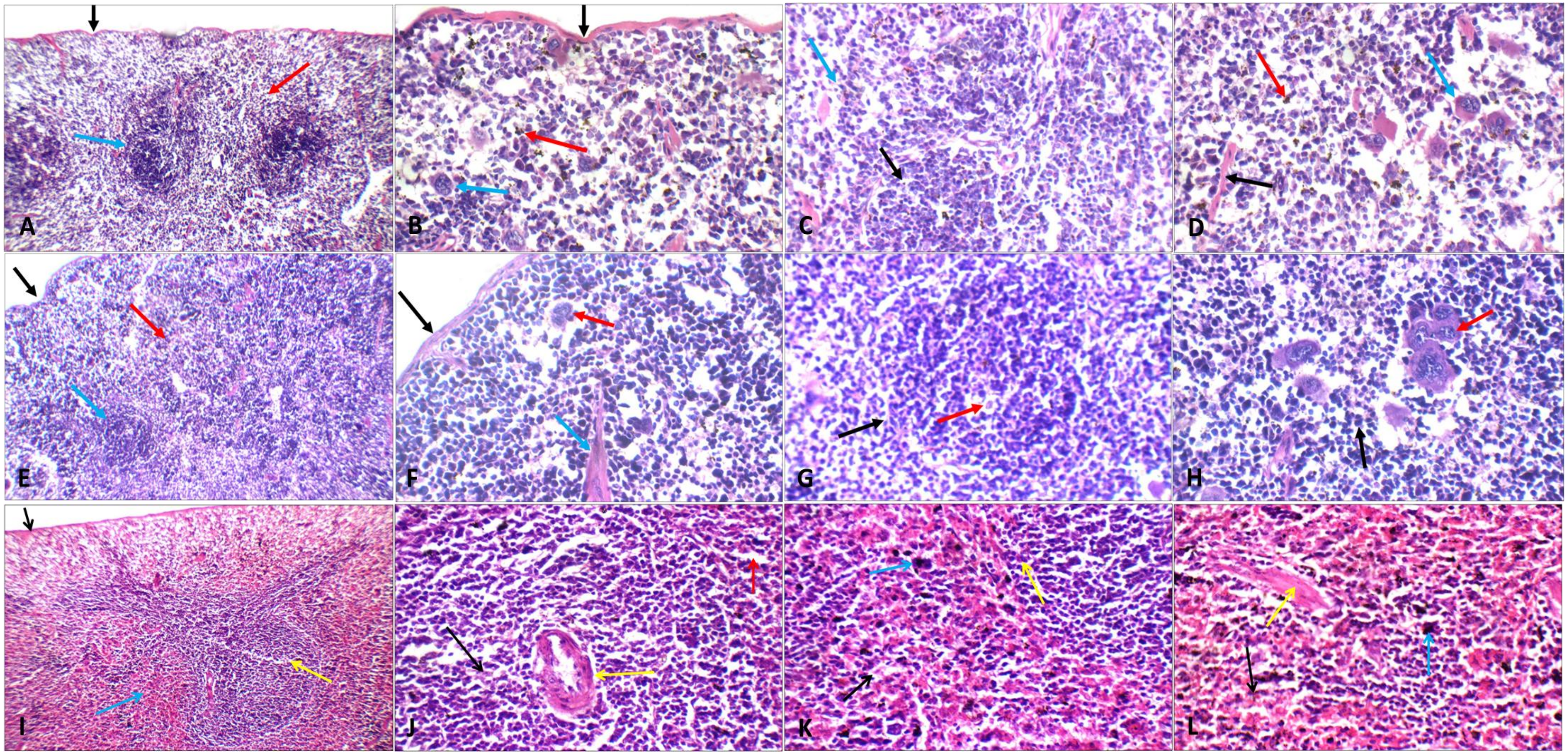

Figure S5: Hematoxylin and eosin rat's spleen sections in:

1- GI (control group) and GII (2.4 mL) showing A] average capsule (black arrow), small-sized lymphoid follicles with hyalinized areas (blue arrow), and expanded red bulb (red arrow) (x200), B] average capsule (black arrow), expanded red bulb with scattered giant cells (blue arrow), and scattered ciderophages (red arrow) (x400), C] small-sized lymphoid follicles with scattered apoptotic lymphocytes in peri-arteriolar area (black arrow), and expanded red bulb (blue arrow) (x400) and D] expanded red bulb with average fibrous septa (black arrow), excess giant cells (blue arrow), and scattered ciderophages (red arrow) (x400).

2- Optimal dose group (GIII) showing E] average capsule (black arrow), small-sized lymphoid follicles (blue arrow), and expanded red bulb (red arrow) (x200), F] average capsule (black arrow), expanded red bulb with average fibrous septa (blue arrow) and scattered giant cells (red arrow) (x400), G] small-sized lymphoid follicles (black arrow), with scattered apoptotic lymphocytes in peri-arteriolar area (red arrow) (x400) and H] expanded red bulb (black arrow), with scattered giant cells (red arrow) (H&E x 400).

3- High doses groups (GIV and GV) showing I] average capsule (black arrow), average-sized well- defined lymphoid follicles (yellow arrow), expanded mildly congested red bulb (blue arrow) (x200), J] average lymphoid follicles with average central arteriole (yellow arrow), scattered apoptotic lymphocytes (black arrow), and mildly congested blood sinusoids (red arrow) (x400), K] lymphoid follicles with scattered apoptotic lymphocytes (yellow arrow), markedly congested blood sinusoids (black arrow), and scattered ciderophages (blue arrow) (x400) and L] average fibrous septa (yellow arrow), markedly congested blood sinusoids (black arrow), and scattered ciderophages (blue arrow) (x400).

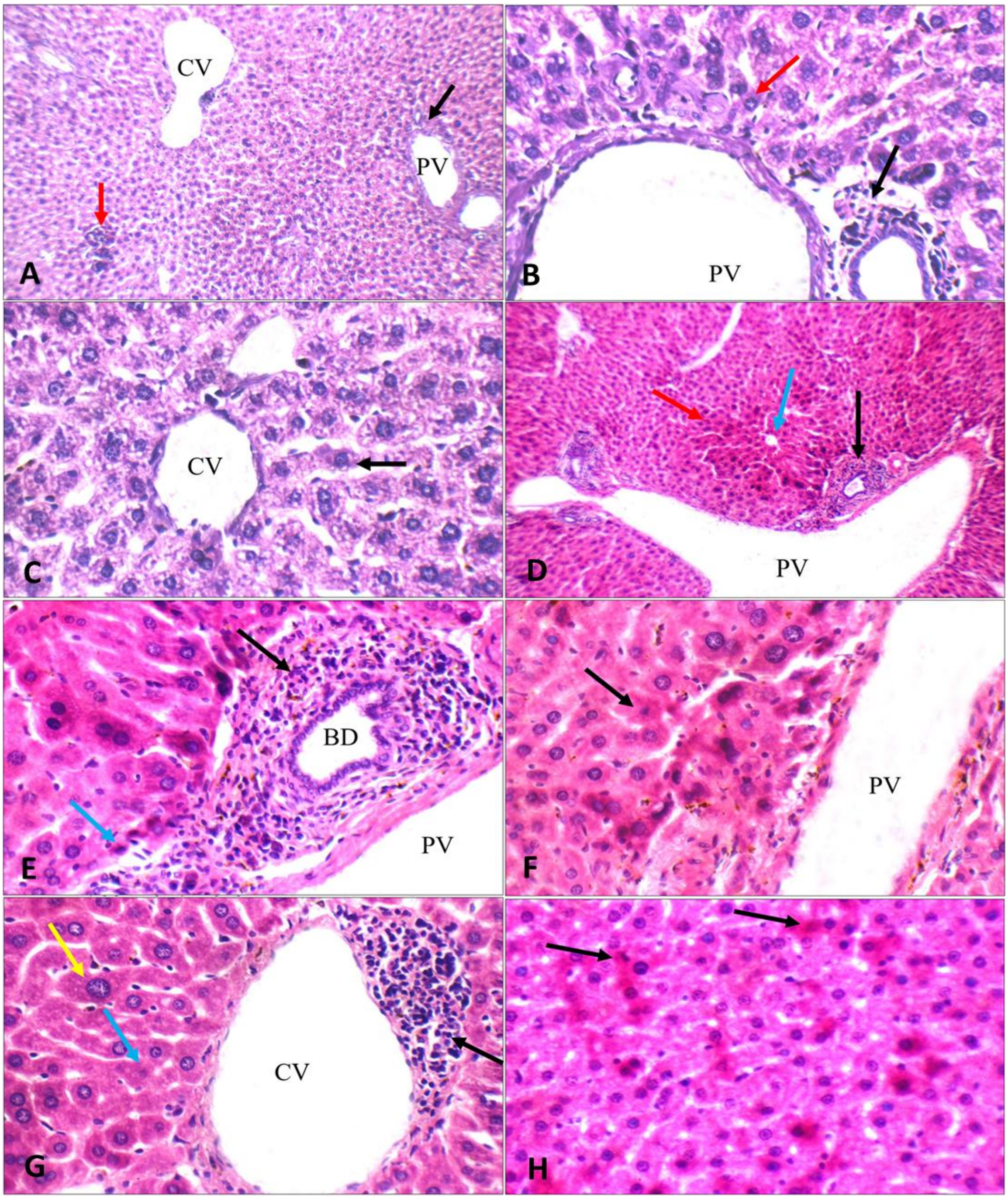

Figure S6: Hematoxylin and eosin rat's liver sections in:

1- GI (control group), GII (2.4 mL) and optimal dose group GIII (3.4 mL) showing A] average portal tracts (black arrow), average portal vein (PV), central vein (CV), and intra-lobular inflammatory infiltrate (red arrow) (X200), B] portal tracts with mild portal inflammatory infiltrate (black arrow), mildly dilated portal vein (PV), and average hepatocytes in peri-portal area (red arrow) (X400), C] average central vein (CV), and average hepatocytes in peri-venular area (red arrow) (X400).

2- high doses groups (GIV and GV) showing D] portal tracts with moderate portal inflammatory infiltrate (black arrow), markedly dilated congested portal vein (PV), average central vein (blue arrow) and markedly apoptotic hepatocytes (red arrow) (X200), E] portal tracts with moderate portal inflammatory infiltrate (black arrow), markedly dilated portal vein (PV), and markedly apoptotic hepatocytes in peri-portal area (blue arrow) (X400), F] portal tracts with markedly dilated portal vein (PV), and markedly apoptotic hepatocytes in peri-portal area (blue arrow) (X400), G] mildly dilated central vein (CV) with moderate peri-venular inflammatory infiltrate (black arrow), and scattered apoptosis (blue arrow) and karyomegally of hepatocytes in peri-venular area (yellow arrow) (X400) and H] markedly apoptotic hepatocytes (black arrow) (X400).

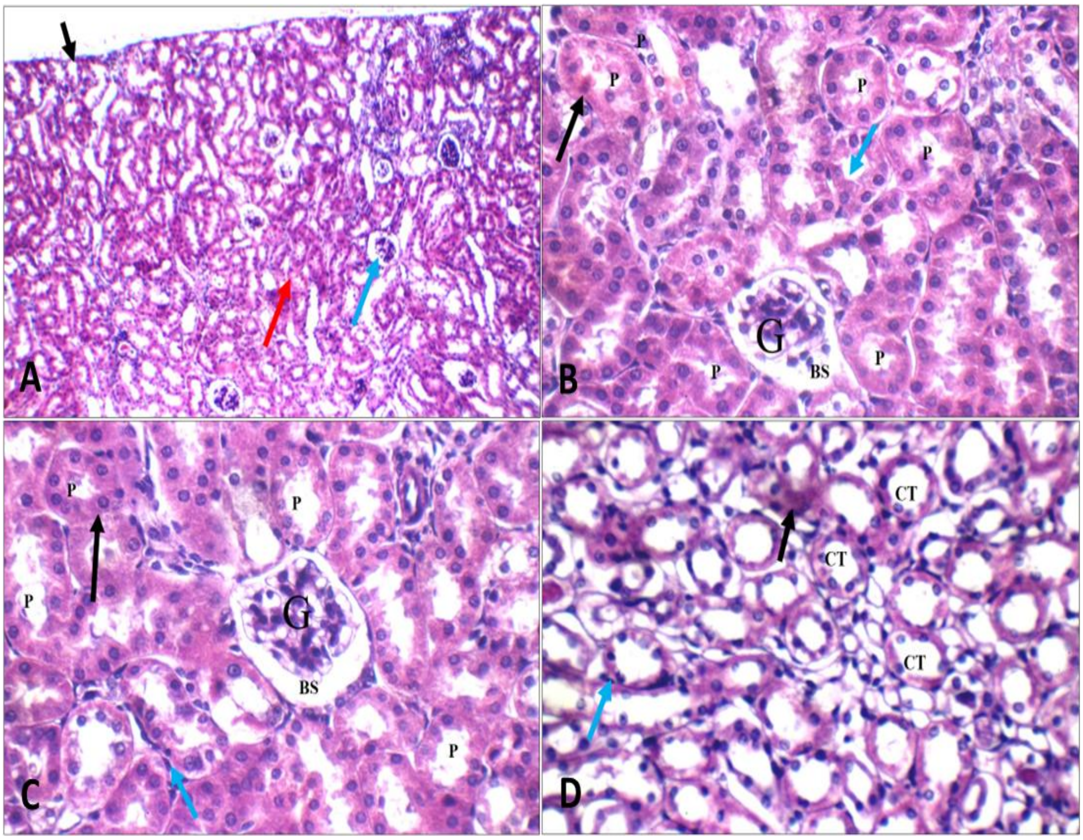

Figure S7: Hematoxylin and eosin rat's kidney sections showing A] average renal capsule (black arrow), average glomeruli (blue arrow), and average tubules (red arrow) (X200), B] average glomeruli (G) with average Bowman's spaces (BS), proximal tubules (P) with scattered apoptotic epithelial lining (black arrow) and preserved brush borders (blue arrow) (X400), C] average glomeruli (G) with average Bowman's spaces (BS), proximal tubules (P) with average epithelial lining (black arrow), and average interstitium (blue arrow) (X400) and D] collecting tubules (CT) with scattered apoptotic epithelial lining (black arrow) and average interstitium (blue arrow) in renal medulla (X400).
